# The *Arabidopsis thaliana onset of leaf death 12* mutation in the lectin receptor kinase P2K2 results in an autoimmune phenotype

**DOI:** 10.1186/s12870-023-04300-0

**Published:** 2023-06-02

**Authors:** Liming Zhao, Hao-Jie Wang, Patricia Dalcin Martins, Joost T. van Dongen, Anthony M. Bolger, Romy R. Schmidt, Hai-Chun Jing, Bernd Mueller-Roeber, Jos H. M. Schippers

**Affiliations:** 1 Institute of Biochemistry and Biology, University of Potsdam, Karl-Liebknecht-Straße 24-25, Haus 20, 14476 Potsdam, Germany; 2 Max Planck Institute of Molecular Plant Physiology, 14476 Potsdam-Golm, Germany; 3 Beijng Academy, Beijing, 100028 China; 4 Department of Molecular Genetics, Leibniz Institute of Plant Genetics and Crop Plant Research (IPK) Gatersleben, 06466 Seeland, Germany; 5 Institute of Biology I, Rheinisch-Westfälische Technische Hochschule Aachen University, 52074 Aachen, Germany; 6 IBG-4: Bioinformatik,Forschungszentrum Jülich, 52425 Jülich, Germany; 7 Plant Biotechnology Group, Faculty of Biology, Bielefeld University, Universitätsstrasse 25, 33615 Bielefeld, Germany; 8 Center for Biotechnology, Bielefeld University, Universitätsstrasse 25, 33615 Bielefeld, Germany; 9 Key Laboratory of Plant Resources, Institute of Botany, Chinese Academy of Sciences, Beijing, 100093 China; 10 University of Chinese Academy of Sciences, Beijing, 100049 China; 11 Center of Plant Systems Biology and Biotechnology (CPSBB), Ruski 139 Blvd, Plovdiv, 4000 Bulgaria

**Keywords:** Arabidopsis, Salicylic acid, Onset of leaf death, Autoimmunity, Receptor-like kinase

## Abstract

**Background:**

Plant immunity relies on the perception of immunogenic signals by cell-surface and intracellular receptors and subsequent activation of defense responses like programmed cell death. Under certain circumstances, the fine-tuned innate immune system of plants results in the activation of autoimmune responses that cause constitutive defense responses and spontaneous cell death in the absence of pathogens.

**Results:**

Here, we characterized the *onset of leaf death 12* (*old12*) mutant that was identified in the Arabidopsis accession Landsberg *erecta*. The *old12* mutant is characterized by a growth defect, spontaneous cell death, plant-defense gene activation, and early senescence. In addition, the *old12* phenotype is temperature reversible, thereby exhibiting all characteristics of an autoimmune mutant. Mapping the mutated locus revealed that the *old12* phenotype is caused by a mutation in the *Lectin Receptor Kinase P2-TYPE PURINERGIC RECEPTOR 2* (*P2K2*) gene. Interestingly, the P2K2 allele from Landsberg *erecta* is conserved among *Brassicaceae*. P2K2 has been implicated in pathogen tolerance and sensing extracellular ATP. The constitutive activation of defense responses in *old12* results in improved resistance against *Pseudomonas syringae pv*. tomato DC3000.

**Conclusion:**

We demonstrate that *old12* is an auto-immune mutant and that allelic variation of *P2K2* contributes to diversity in Arabidopsis immune responses.

**Supplementary Information:**

The online version contains supplementary material available at 10.1186/s12870-023-04300-0.

## Background

Leaf senescence is a kind of programmed cell death (PCD), that is induced by developmental cues and numerous environmental signals to promote the salvage of nutrients from leaves to other organs of the plant, in particular developing seeds [1, 2]. Leaf senescence is an active genetically controlled process and typified by the upregulation of senescence associated genes (SAGs) [3]. The ordered dismantling of the chloroplast during senescence gives rise to the typical color changes of leaves and is one of the main processes that provides nutrients for sink tissues [4]. Initial genetic studies of senescence mutants have revealed that the onset of leaf death is balanced by the action of senescence-promoting and preventing genes [5–8]. Currently, numerous molecular components have been identified that affect the onset of leaf senescence. As these factors participate in different cellular, hormonal, metabolic or transcriptional pathways it has become evident that there is a complex control of leaf senescence that involves multiple molecular components and regulatory pathways [2].

Developmental senescence typically results in the upregulation of defense-related genes that are typically induced upon pathogen attack [9, 10]. Although PCD is triggered both during senescence and pathogen infection, the processes are different as pathogens invoke a hypersensitive response (HR) that first results in local cell death [11]. Still, in both cases salicylic acid (SA) levels as well as the expression of pathogenesis-related (PR) genes increase [12]. Moreover, repression of SA levels both delays pathogen-induced PCD as well as developmental senescence. Loss of SENESCENCE-ASSOCIATED E3 UBIQUITIN LIGASE 1 (SAUL1) has been shown to result in a premature senescence phenotype [13], that relies on the PHYTOALEXIN DEFICIENT 4 (PAD4)-dependent SA pathway [14]. In addition, it was found that SAUL1 integrates signals from temperature- and humidity-dependent defense programs with the onset of leaf senescence [15]. Moreover, the *saul1* mutant exhibits characteristics of an autoimmune mutant with improved tolerance against the bacterium *Pseudomonas syringae pv*. tomato DC3000 (*Pst* DC3000). Therefore, the *saul1* mutant represents an example of how related defense and senescence pathways are, which both in the end result in PCD. Of note, *Pst* DC3000 is a well-known hemibiotrophic pathogen that originated from tomato and encodes almost 40 Hop effectors (Hypersensitive response and pathogenicity outer proteins), which are not recognized by R proteins in Arabidopsis making it susceptible to *Pst* DC3000 [16, 17]. During senescence, SA levels increase but are strictly controlled by SAG108, an SA 3-hydroxylase in *Arabidopsis thaliana* [18]. Loss of *SAG108* results in increased SA levels and precocious senescence, while overexpression delays the onset of senescence, suggesting that SA levels control the onset and rate of leaf senescence [18]. Potentially, the fine-tuning of SA levels during senescence and pathogen responses determines the kind of PCD induced.

Plants get exposed to a variety of biotic stresses during their life cycle, which requires an appropriate immune response to ensure their survival [19]. During evolution, plants acquired two types of defense responses. The first is the so-called pattern-triggered immunity (PTI) response [20], which is triggered by the detection of pathogen associated molecular patterns (PAMPs) via membrane‐localized pattern recognition receptors (PRRs). The activation of PTI results, amongst others, in the enhanced expression of defense‐related genes, callose deposition and growth inhibition. In addition, pathogens introduce effector proteins into their hosts, which are directly or indirectly detected by nucleotide‐binding site leucine‐rich repeat (NBS‐LRR)‐type resistance proteins (NLRs) and induce so-called effector-triggered immunity (ETI) [19]. Plant proteins that recognize pathogen effectors, so-called R-proteins, typically activate defense‐related gene expression, the HR response and affect plant growth [21]. As the activation of plant defense responses can cause growth defects, plants continuously need to balance the trade-off between growth and immunity responses [22, 23]. Interestingly, recent advances in immunity research have shown that dichotomies between PTI and ETI are often blurred [24]. To overcome this, the spatial immunity model was proposed, that differentiates immunity based on the location of the danger signal. Detection of this signal occurs either extracellular, through a so-called extracellular immunogenic pattern (ExIP), or intracellular by an intracellular immunogenic pattern (InIP).

Mutants that constitutively accumulate SA, like *constitutive expression of PR genes 1* (*cpr1*), *cpr5*, *cpr6* [25, 26], and *accelerated cell death 6* (*acd6*) and *acd11* [27, 28], not only have HR-like phenotypes, but are also more resistant to pathogens due to the activation of the defense response. The aforementioned mutants are known as autoimmune mutants exhibiting hallmarks of innate immune responses, such as marker gene induction and accumulation of the defense hormone SA. Furthermore, these plants are commonly dwarfed, and they show tissue necrosis and spontaneous cell death [29]. Autoimmunity in Arabidopsis has been observed in plants ectopically expressing proteins (e.g. ENHANCED DISEASE SUSCEPTIBILITY1 (EDS1) and the basic helix-loop-helix-type 84 (bHLH84) transcription factor [30]), in forward genetic screens, and in crosses between different accessions (hybrid incompatibility). The underlying molecular cause for autoimmune responses is often due to NLRs that recognize the aberrant or accession-foreign protein isoform [31, 32].

Previously, we have isolated the *onset of leaf death 12* (*old12*) mutant from an ethyl methanesulfonate (EMS)-induced Arabidopsis Landsberg *erecta* (L*er*-0) mutant population based on its early senescence phenotype [6]. Here, we characterize the *old12* mutant and reveal that it represents a typical autoimmune mutant. The observed growth retardation and precocious cell death occur in a temperature- and SA-dependent manner. Next-generation sequencing of the mutant genome revealed a point mutation in the *Lectin Receptor Kinase (LecRK) P2-TYPE PURINERGIC RECEPTOR 2* (*P2K2*) gene; the protein it encodes has been implicated in sensing extracellular ATP (eATP) [33]. Upon developmental stimuli or during stress, plant cells release ATP from the cytosol to the extracellular matrix, whereby the eATP functions as a signaling molecule [34]. During mechanical or biotic stress eATP serves ExIP [33]. We found that the OLD12 point-mutation converts a conserved cysteine residue to a tyrosine (C407Y), which impairs kinase activity. In addition, the constitutive upregulation of defense responses results in improved resistance against *Pst* DC3000. Furthermore, this study reveals natural variation in the functioning of P2K2 in Arabidopsis.

## Results

### *old12* mutant shows early onset of leaf death and spontaneous lesion formation

**Fig. 1:**
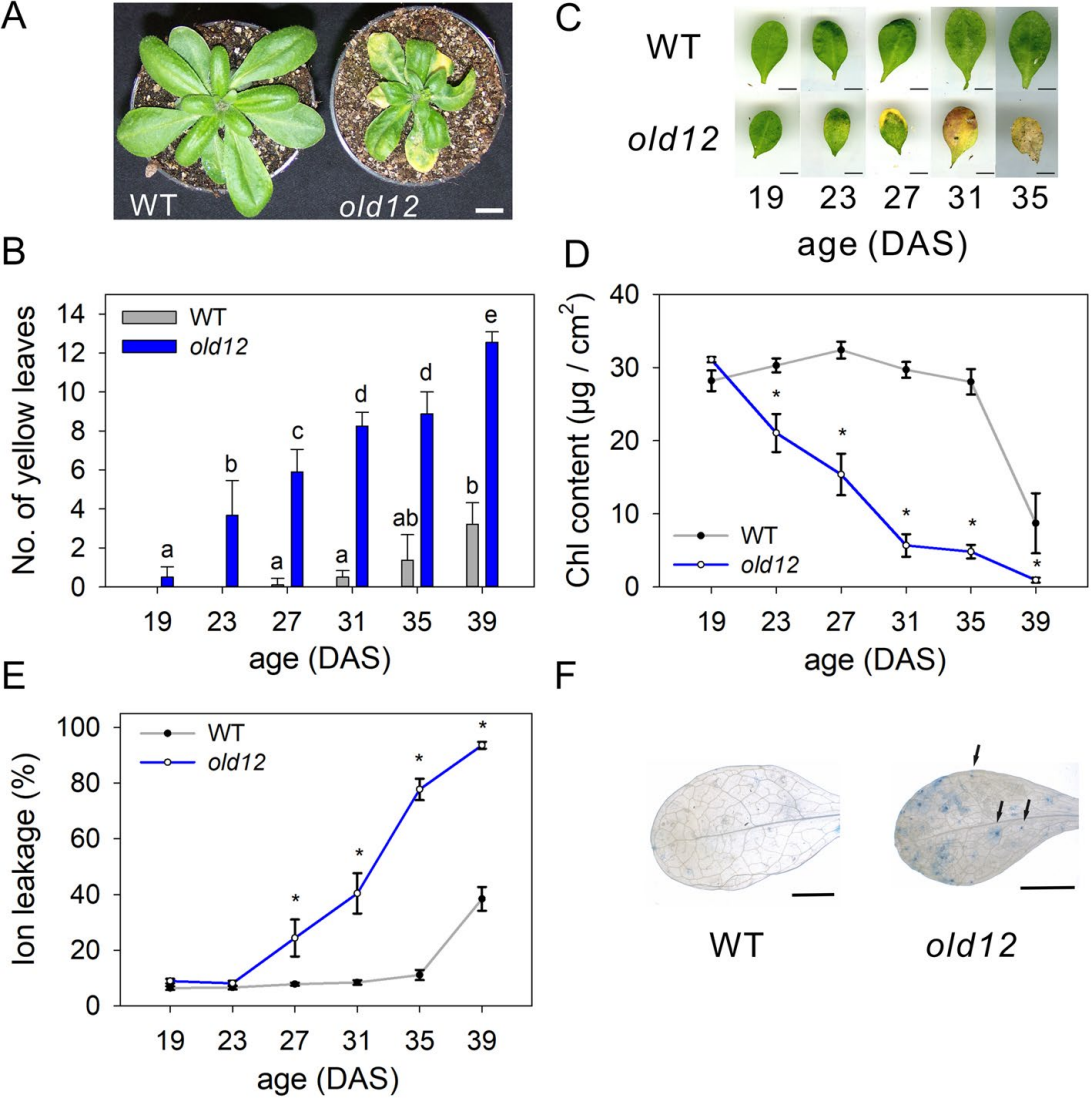
The early cell death phenotype of *old12*. **A** Image of 4-week-old *Arabidopsis thaliana* wild-type (WT) and *old12* plants grown in long-day photoperiod (16 h). Scale bar = 1 cm. **B** Number of yellow (/senescent) leaves at different time points after the sowing of wild-type plants and *old12*. Each data point represents the means of three replicates of ten plants each ± SD. Different letters indicate significant difference according to one-way ANOVA and post-hoc Tukey HSD test. **C** Progression of senescence and cell death in the second leaf pair of *old12* and wild type from 19 to 35 DAS (days after sowing). Scale bar = 1 cm. **D**, **E** Chlorophyll (Chl) content and ion leakage in the second leaf pair of *old12* and wild type (WT) from 19 to 39 DAS. Each data point represents the mean ± SD from three replicates of six leaves each. Asterisk indicates significant difference (*p* ≤ 0.05; Student´s *t*-test) between WT and *old12* at the indicated time points. **F** Cell death staining (trypan blue) of the third leaf of wild-type and *old12* leaves at 23 DAS. Arrows indicate lesions. Scale bar = 5 mm

The *old12* mutant was originally isolated from an EMS-mutagenized Arabidopsis population in the accession L*er*-0 [26]. The *old12* phenotype segregates as a recessive trait and the onset of leaf death is not enhanced by exogenously applied ethylene [6]. Under long-day conditions (photoperiod length of 16 h), the *old12* mutant develops like the corresponding Arabidopsis wild type during the first two weeks of cultivation; however, an early senescence and cell death phenotype was observed thereafter (Fig. 1A, B). This phenotype was also observed at a photoperiod length of eight hours (short-day condition; Fig. S1A). Of note, the *old12* mutant does not show any altered germination from the wild type (Fig. S1B). The second leaf pair of *old12* showed a decline in chlorophyll levels from 23 days after sowing (DAS) onwards while in the wild type this was first observed at 35 DAS (Fig. 1C, D). The early loss of cell vitality in *old12* was demonstrated by an increase in ion leakage from 27 DAS onwards which in the wild type occurs first at 39 DAS (Fig. 1E). Usually, age-dependent senescence starts at the tip of the leaf, slowly progresses towards the base, and ends with controlled cell death [1, 18]. Leaves of *old12* show typical yellow/brown spots prior to whole-leaf senescence, indicating potential premature lesion formation. To confirm the occurrence of cell death, a trypan blue staining was performed on the third leaf of 23 DAS-old plants (Fig. 1F). The *old12* leaves showed distinct trypan blue-stained spots over the entire leaf blade while this was not observed in wild type. The observed spots are characteristic for lesion-mimic mutants like *acd* and *lesion simulating disease* (*lsd*) that spontaneously activate PCD and defense responses [28, 35].

### The *old12* phenotype is temperature dependent

**Fig. 2:**
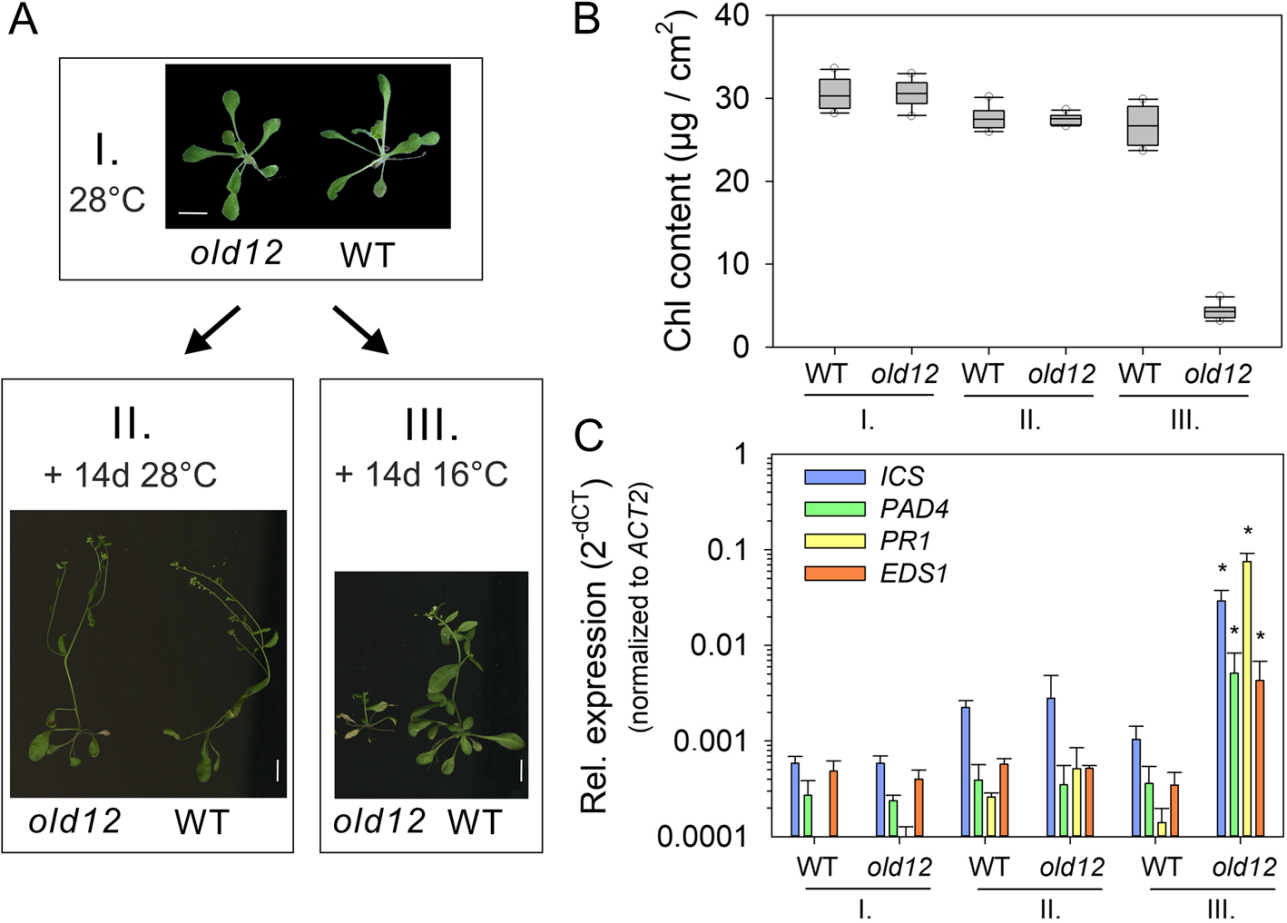
The *old12* mutant displays a low-temperature-induced lesion mimic phenotype.** A** Panel I, 28 DAS *old12* and wild-type (WT) plants grown in long-day photoperiod (16 h) at 28 °C. Panel II, Phenotype of *old12* and WT plants grown for 28 days + 14 days at 28 °C. Panel III, Transfer of *old12* plants grown for 28 days at 28 °C to 16 °C for 14 days results in stunted growth and an early onset of senescence as compared to WT. Scale bar = 1 cm. **B** Chlorophyll (Chl) content in the fifth and sixth leaf of *old12* and WT at the different growth conditions shown in (**A**). Box plots show median and interquartile ranges (IQR), outliers (> 1.5 times IQR) are shown as circles. **C** Expression of SA pathway and defense-related genes in *old12* and WT leaves grown for 28 days at 28 °C (I), or 28 days at 28 °C plus 2 days at 28 °C (II), or 28 days at 28 °C plus 2 days at 16 °C (III). The relative expression level is shown as mean ± SD of three biological replicates. Expression level for each time point was normalized against *ACTIN2* (*ACT2*). Asterisks indicate significant difference under the conditions tested between *old12* and wild type for the genes indicated (Student’s *t*-test; *p* ≤ 0.05)

To date, a large number of lesion mimic mutants have been identified and characterized that helped to unravel the molecular pathways regulating PCD in plants [36]. The hypersensitive response is usually connected with pathogen resistance, but can also cause pathogen susceptibility and growth retardation when inappropriately activated. Interestingly, HR induced by most plant–pathogen interactions and auto-active NLRs is frequently temperature sensitive such that HR is suppressed at temperatures above 20–30 °C [36, 37]. Moreover, *old12* belongs to the same class of old mutants as *old3*, which was previously shown to have a temperature-dependent phenotype [38]. Cultivation of *old12* at 28 °C for 27 days resulted in a fully recovered phenotype indistinguishable from wild type (Fig. 2A). Subsequently, 18 plants were cultivated further for 14 days at 28 °C while another 18 plants were further cultivated at 16 °C. The *old12* plants kept at 28 °C remained indistinguishable from wild type and developed normally, however, those transferred to 16 °C showed a severe growth reduction and an early onset of senescence and cell death.

Measurement of chlorophyll content of *old12* mutants and wild-type plants grown at elevated temperature revealed no significant difference (Fig. 2B). Yet, *old12* plants transferred to cold conditions had a clear decrease in chlorophyll levels due to onset of senescence. Typical lesion mimic or autoimmune mutants show activation of immune responses at the transcript level, including SA pathway and defense-related genes [39]. Here, the expression of *ISOCHORISMATE SYNTHASE 1* (*ICS1*), *PHYTOALEXIN-DEFICIENT 4* (*PAD4*), *PATHOGENESIS-RELATED PROTEIN 1* (*PR1*) and *EDS1* was determined in both *old12* and the wild type under all growth conditions (Fig. 2C). Of note, gene expression after the transfer to cold conditions was tested after 48 h. At 28 °C, expression of the selected genes was similar between *old12* and wild type (condition I and II). In contrast, transfer of *old12* to 16 °C resulted in a strong induction of all SA-related genes. These results imply that *old12* is an autoimmune mutant, triggering defense responses at low temperature resulting in spontaneous cell death and early senescence.

### *OLD12* encodes the lectin receptor kinase P2K2

Genetic analysis of backcrossed *old12* with wild-type plants indicated that the mutant harbors a monogenic recessive mutation in a nuclear gene [6]. To generate a mapping population, the *old12* mutant (L*er*-0 background) was crossed with the accession Col-0. Segregation analysis of the *old12* phenotype in the mapping population revealed a 15:1 ratio (green:yellow), suggesting that the phenotype depends on two loci in the mapping population (Table 1). To gain more insight into the impact of the Arabidopsis background on the segregation of the phenotype, the *old12* mutant was backcrossed with Ct-1, Nd-0, Tsu-0 and C24. Backcrosses with Nd-0 or C24 resulted in a 3:1 segregation, while backcrosses with Ct-1 and Tsu-0 revealed a 15:1 ratio, similar as observed for Col-0. The data suggest that one main locus causes the *old12* phenotype but in several accessions, a second locus is required for the phenotype.

**Table 1:** Genetic segregation of the early senescence phenotype in F_2_ populations between *old12* and several different Arabidopsis accessions

F_2_ population	G	Y	Exp. Ratio (G:Y)	No. of loci	χ^**2**^ (* *P* < 0.05)
*old12* x Ler-0	96	33	3:1	1	0.02*
*old12* x Col-0	814	42	15:1	2	2.63*
*old12* x Ct-1	144	8	15:1	2	0.25*
*old12* x Nd-0	92	19	3:1	1	3.67*
*old12* x Tsu-0	102	6	15:1	2	0.09*
*old12* x C24	87	20	3:1	1	2.27*

**Fig. 3:**
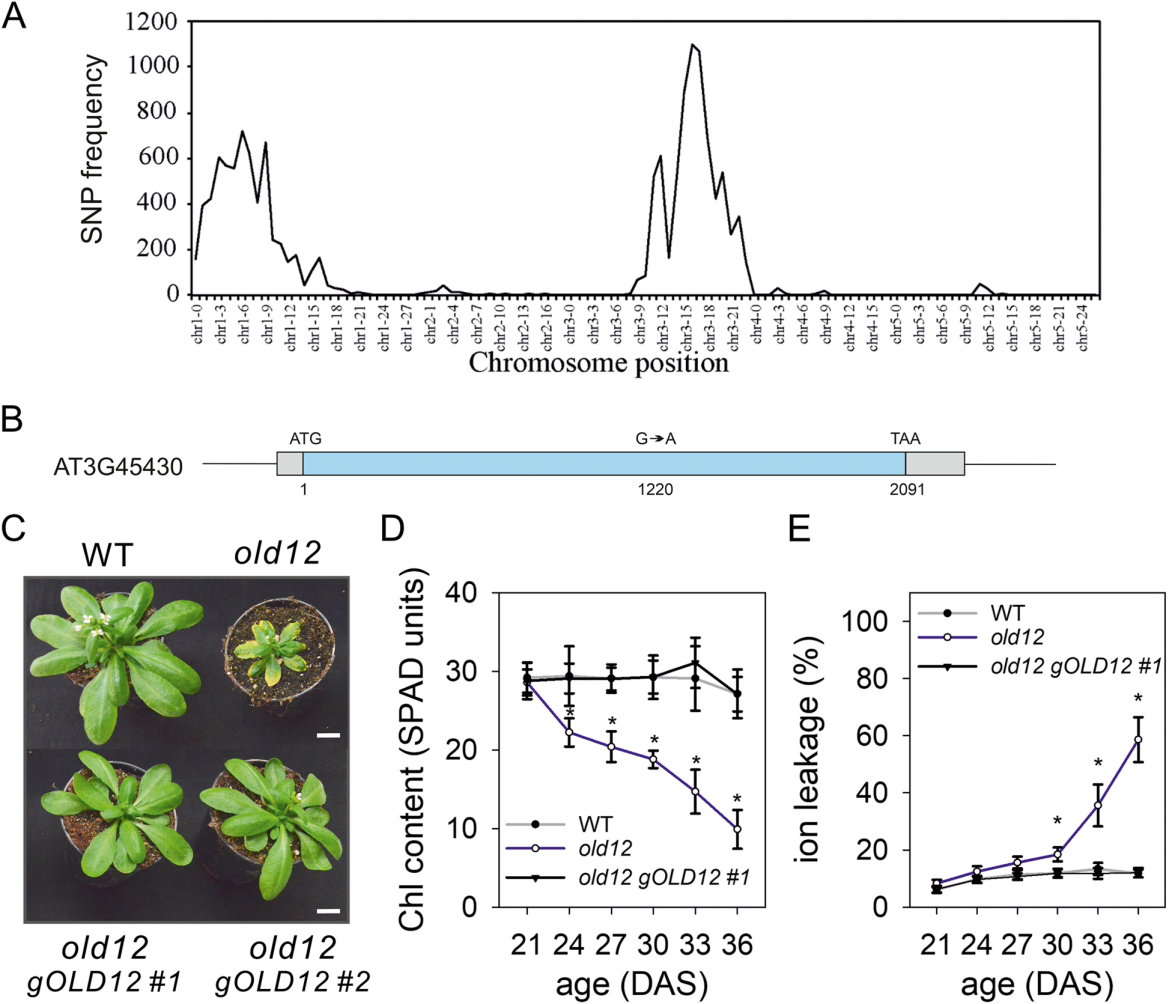
Identification of the *old12* mutation by whole-genome sequencing. **A** Genome-wide SNP frequency plot of the Arabidopsis genome using a bin size of 1 Mb for the *old12* x Col-0 mapping population. Two candidate regions were identified for the *old12* locus, one on chromosome I and one on chromosome III. **B** Genomic representation of the point mutation identified in *P2K2* (AT3G45430). Light blue indicates exon sequence and gray corresponds to untranslated regions. **C** 32 DAS wild type (WT), *old12* and two independent complementation (*old12 gOLD12*) lines grown under long-day photoperiod (16 h). Complementation of *old12* with the genomic region containing AT3G45430 restores the wild-type phenotype. Scale bar = 1 cm. **D** Chlorophyll content (SPAD units) of the 3rd/4th leaf from WT, *old12* and *old12 gOLD12 #1*. Each data point represents the mean ± SD from 10 leaves. **E** Ion leakage of the 3rd/4th leaf from WT, *old12* and *old12 gOLD12 #1*. Each data point represents mean ± SD from 6 replicates of three leaves each. Asterisks indicate significant difference from WT (Student’s *t*-test; *p* ≤ 0.05)

To identify the responsible EMS mutation, the genomic DNA of a pool of 157 F_2_ *old12 x* Col-0 plants displaying the phenotype was isolated and sequenced. We used both SHOREmap and next generation mapping (NGM) to analyze the obtained data [40, 41]. Plotting of the identified sequence variance with SHOREmap resulted in two clusters, one on the left arm of chromosome I and one on the right arm of chromosome III (Fig. S2). By using the NGM tool we were able to narrow down the mapped region on chromosome I to a 4 Mb region and for chromosome III to a 3 Mb region (Fig. 3A). Single-nucleotide polymorphisms (SNPs) corresponding to an EMS mutation (G to A or C to T) were called against the reference genome Col-0, resulting in seven loci with a putative hit for chromosome I (Table S1). However, all identified SNPs turned out to be due to natural variation between the two accessions [42], suggesting that on chromosome I no gene was affected by the EMS mutagenesis. In contrast, the region on chromosome III contained 50 SNPs, of which one was not due to accession differences but represented the potential mutation (G to A) causing the phenotype due to a hit in AT3G45430, encoding the *L-TYPE LECTIN RECEPTOR KINASE I.5* (*LecRK-I.5*) / *P2-TYPE PURINERGIC RECEPTOR 2* (*P2K2*; Fig. 3B).

To confirm that the mutation in *P2K2* is indeed causative for the *old12* phenotype, a complementation of the *old12* mutant with a genomic fragment from L*er*-0 (*gOLD12*), spanning a 2-kb promoter region, the coding region, and a 500-bp downstream sequence was performed. More than ten independent T1 complementation lines were obtained. Selected T2 complementation lines showed a full reversal of the *old12* phenotype (Fig. 3C) as indicated by a wild-type phenotype and the absence of early leaf senescence and death (Fig. 3D, E). These results clearly demonstrate that the *old12* phenotype is caused by the point mutation in *P2K2*. Next to a complementation assay, we also isolated a homozygous T-DNA insertion line in the Col-0 background (*p2k2-1*). However, loss-of-function mutant plants of *P2K2* did not result in the early leaf senescence phenotype (Fig. S3), which is in line with the observed genetic dependence of the OLD12 phenotype on a second locus on chromosome I.

### The *old12* mutation impairs kinase activity of P2K2

**Fig. 4:**
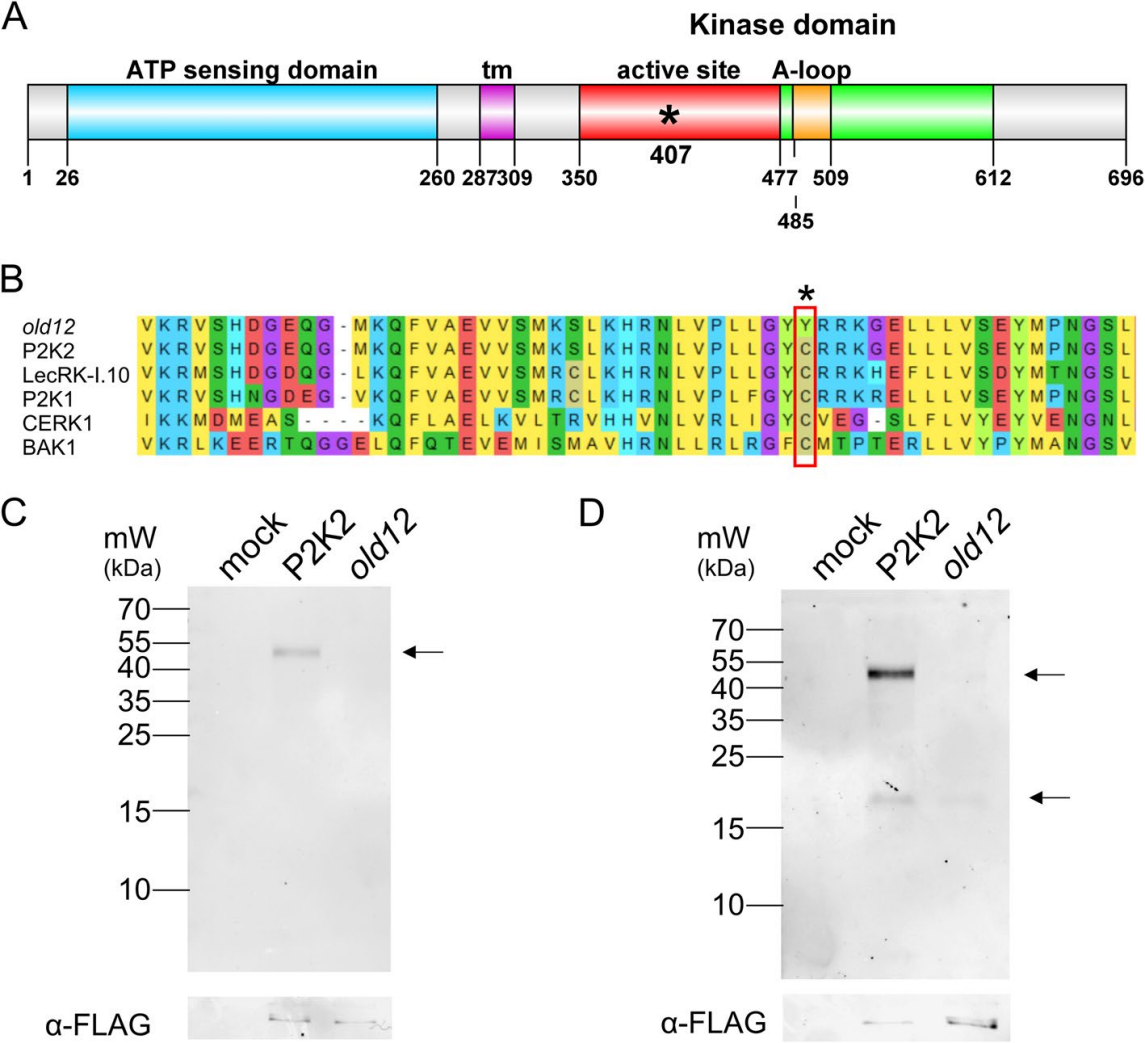
The *old12* mutation impairs P2K2 kinase activity. **A** Schematic representation of the P2K2 protein structure highlighting the different domains and the position of the *old12* mutation (C407Y, asterisk). **B** Multiple sequence alignment of the active site of several LecRK proteins and other receptor like kinases (RLKs). The *old12* mutation affects a cysteine residue that is conserved in the kinase domain. **C** P2K2 autophosphorylation activity was analyzed using an expressed and purified FLAG-tagged kinase domain (KD) of the wild-type P2K2 and the *old12* mutant. Autophosphorylation was measured by detecting thiophosphorylation with ATPγS and subsequent western blotting using thiophosphate ester-specific antibodies. **D** Transphosphorylation assays were performed by incubating purified FLAG-tagged KDs of wild-type P2K2 and *old12* with the universal kinase substrate MBP. Thiophosphorylation of MBP was subsequently detected by a western blot using antibodies against the thiophosphate ester. The upper arrow indicates autophosphorylation of P2K2, while the lower arrow indicates the transphosphorylation of MBP. Mock reactions only contained the MBP substrate. The loading control involves the detection of OLD12 proteins with an anti-FLAG antibody. Images for the loading controls are cropped, full images can be found in the supplement (Fig. S6)

P2K2 has previously been identified as an extracellular ATP-sensing kinase that can complement the function of LecRK-I.9/P2K1 [33, 34]. The protein contains an extracellular ATP-sensing domain, a transmembrane region, and a cytoplasmic kinase domain (Fig. 4A). Of note, in L*er*-0, the cytosolic domain is 22 amino acids longer than in Col-0 (Fig. S4). In addition, in C24 the *old12* mutant phenotype relies only on the *P2K2* gene, however, also the C24 protein does not have the C-terminal extension (Fig. S4) [43]. Moreover, the cytosolic C-terminal extension of P2K2 was found amongst different *Brassicaceae* (Fig. S5), including radish, rape, mustard and shepherd's-purse, indicating that it is a conserved feature of P2K2.

The *old12* mutation is found in the cytosolic kinase domain, changing a cysteine residue into a tyrosine (C407Y). Subsequently, a sequence comparison of the active site region from *P2K2* with that of other receptor-like kinases was made to understand the potential impact of the mutation (Fig. 4B). Here, especially the kinase domains of different membrane proteins with a known role in plant immunity were selected to exemplify that the amino acid residue mutated in *old12* is present in key proteins of plant immunity. The C407 residue from P2K2 is highly conserved and found not only in other LecRK proteins (P2K1 and LecRK-I.10), but also in BRI1-ASSOCIATED RECEPTOR KINASE (BAK1) and CHITIN ELICITOR RECEPTOR KINASE 1 (CERK1). The position of the mutation suggests that potentially the kinase activity of P2K2 is impaired by the *old12* mutation. LecRKs are kinases with an aspartate (D) in the catalytic loop preceded by a conserved arginine (R) and require auto-phosphorylation of the activation loop for full kinase activity [44, 45].

In order to assess the impact of the *old12* mutation on the P2K2 kinase activity, the cytosolic kinase domain was cloned, purified and used in an autophosphorylation and trans-phosphorylation assay. In comparison to wild-type P2K2, the *old12* mutation results in an impairment of autophosphorylation activity in vitro (Fig. 4C). In addition, the ability to trans-phosphorylate myelin basic protein (MBP) by P2K2 was lost by the *old12* mutation, indicating an impaired kinase activity (Fig. 4D).

### *P2K2* is mainly expressed in root tips

**Fig. 5:**
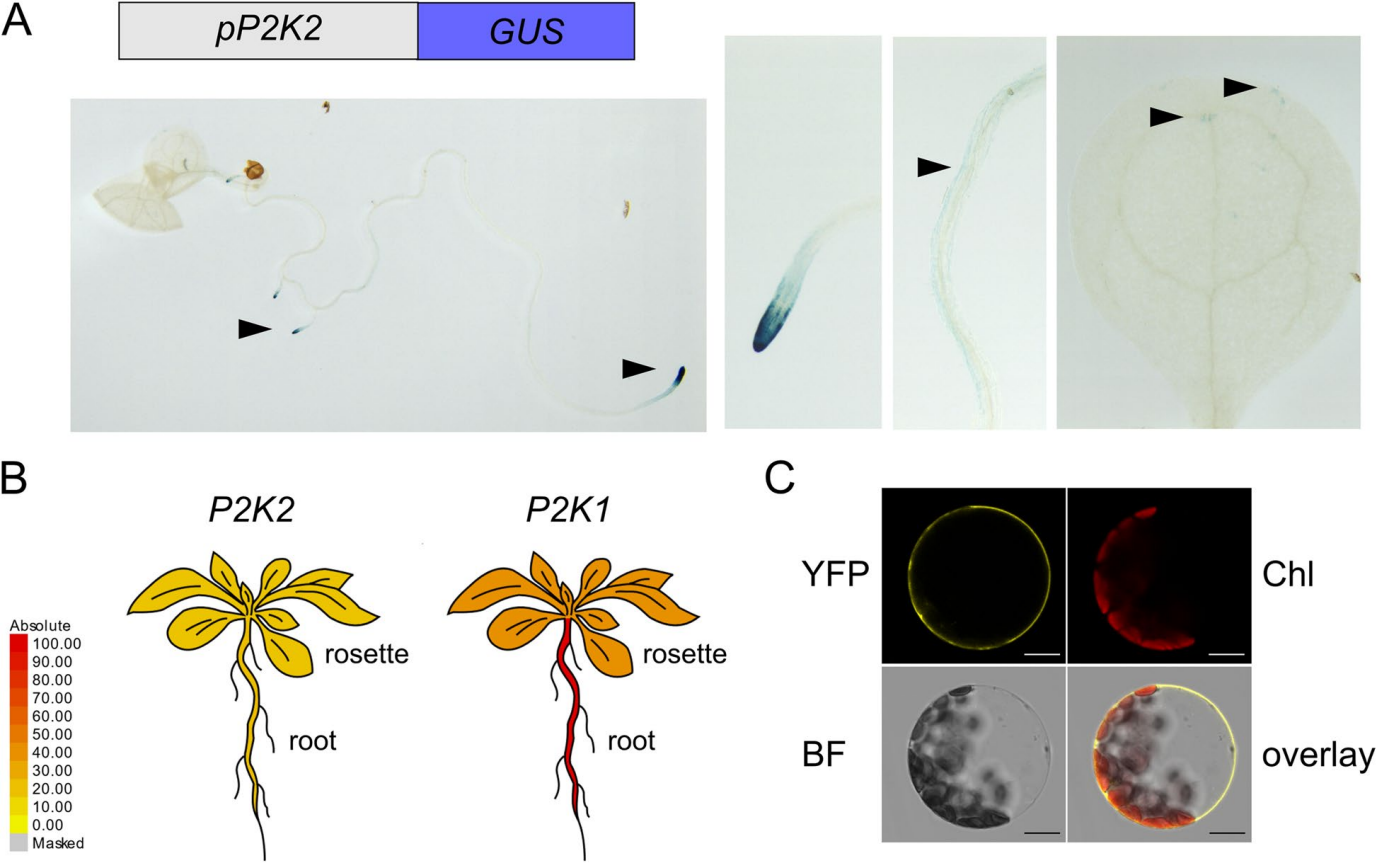
Tissue-specific expression pattern of *P2K2*. **A** The expression pattern of *P2K2* was analyzed using a GUS reporter driven by a 2.1-kb promoter of *P2K2*. Two-week-old plants were analyzed. Arrowheads indicate detection of expression in root tips, root epidermis layer and leaf. **B** As compared to *P2K1*, *P2K2* is weaker expressed, suggesting that P2K1 might be the main extracellular ATP sensing kinase. The image was created with the eFP2 browser (http://bar.utoronto.ca/efp2/Arabidopsis/Arabidopsis_eFPBrowser2.html). **C** Subcellular localization of P2K2-YFP in Arabidopsis mesophyll protoplasts. YFP fluorescence was observed at the plasma membrane. Chl: chlorophyll autofluorescence; BF: bright field. Scale bar = 15 µm

Among the 45 *LecRK* genes in Arabidopsis, only five appear to be highly and broadly expressed under non-stress growth conditions [46]. To reveal the tissue-specific expression pattern of *P2K2,* the upstream promoter sequence of 2.1 kb was used to drive a *GUS* reporter gene in the L*er*-0 background. The expression of *P2K2* appears to be mainly confined to the root tip of both the main and lateral roots (Fig. 5A). In addition, a weak expression was observed in root and leaf epidermal cells. We obtained a similar result with the promoter derived from the Col-0 allele in the Col-0 background (Fig. S7). Previously, it was shown that P2K2 and P2K1 are both capable of sensing extracellular ATP [33]. A comparison of their expression levels suggests that *P2K1* is ubiquitously present while *P2K2* is weakly expressed (Fig. 5B). As reported before [33], the P2K2 protein localizes to the plasma membrane (Fig. 5C). Since the *old12* phenotype is temperature-dependent, it is of interest to note that *P2K2* expression is not affected by cold or heat stress [47, 48].

### *old12* is an auto-immune mutant that relies on salicylic acid

**Fig. 6:**
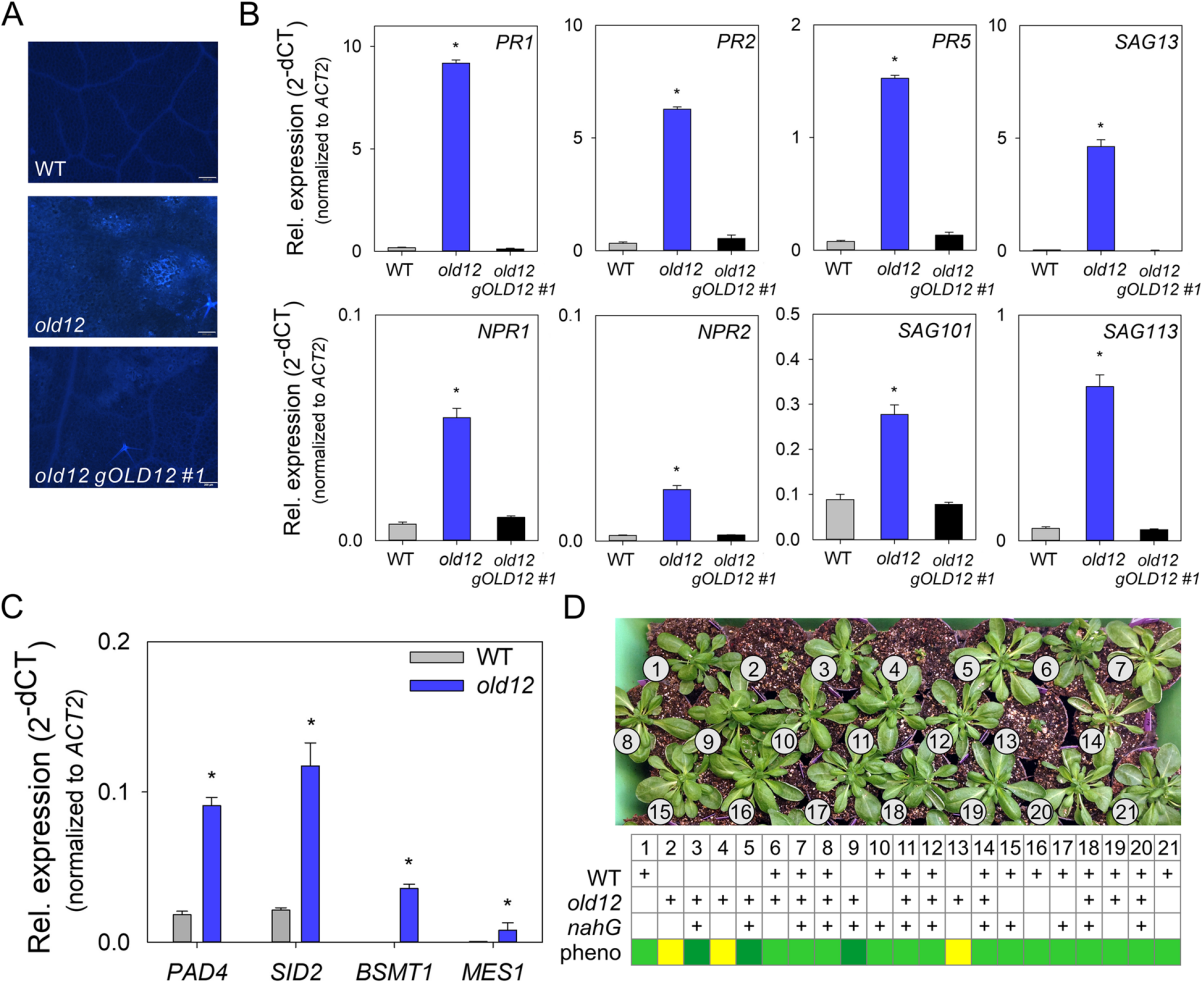
The *old12* auto-immunity phenotype relies on SA. **A** Callose depositions as visualized by aniline blue staining (light blue fluorescence) of 3rd and 4th leaves of 24 DAS (long-day) plants. Scale bar = 200 µm. **B** *PR* gene expression in 27 DAS plants grown on soil under long-day condition. Expression data represents means ± SD from three biological replicates. Expression level for each gene was normalized against *ACT2*. Asterisks indicate significant difference from WT (Student’s *t*-test; *p* ≤ 0.05). **C** Expression of SA biosynthesis genes in L*er*-0 and *old12* mutants. Bars represent mean ± SD from three biological replicates. Asterisks indicate significant difference from WT (Student’s *t*-test; *p* ≤ 0.05). **D** Crossing *nahG* into the *old12* mutant background reverts the early-senescence phenotype. An F2 population of *nahG* x *old12* was phenotyped and genotyped to determine the presence of *old12* SNPs, and the presence of the *nahG* gene. In the table, a ´ + ´ indicates the presence of either the wild-type and/or *old12* allele, and/or the *nahG* gene. Early senescence is indicated by a yellow score in the table, a green score indicates no early senescence, whereby a dark green color highlights plants that are homozygous for the *old12* mutation and at the same time contain the *nahG* transgene. Shown are plants grown under long-day conditions at 28 DAS

As shown above (Figs. 1 and 2), *old12* shows spontaneous lesion formation and increased expression of SA pathway- and defense-related genes. The hypersensitive response causing cell death is often accompanied by callose apposition at cell walls [49]. To test if o*ld12* leaves indeed possess altered callose deposition, a staining with aniline blue was performed (Fig. 6A). Compared to wild type, *old12* leaves displayed a clearly increased deposition of callose, which was no longer observed after complementation.

To further characterize the *old12* mutant we tested the expression of typical defense-related genes in the absence of biotic stress. The PR family covers a group of heterogeneous genes that have been widely used as molecular markers in plant defense responses [50]. Expression of *PR1*, *PR2* and *PR5* was found to be upregulated in the *old12* mutant as compared to wild type (Fig. 6B). In addition, several SAG genes that are also implemented in plant defense responses [51, 52] are upregulated in *old12* plants, including *SAG13*, *SAG101* and *SAG113*. Salicylic acid (SA) is necessary for plant defense against some pathogens, and the perception of this hormone requires NPR1 [53]. Here, we found that both *NPR1* and *NPR2* are upregulated in *old12* mutant plants as compared to the wild type. Of note, upregulation of defense response genes is reverted in the complementation line. The constitutive upregulation of immune-related genes in *old12* plants is characteristic for auto-immune mutants.

Autoimmunity in plants is often associated with activation of the SA signaling pathway [37]. To understand if SA biosynthesis and signaling in the *old12* mutant is altered, we analyzed the expression of several genes. The SA biosynthesis genes *METHYLESTERASE 1* (*MES1*) and *SALICYLIC ACID INDUCTION DEFICIENT2* (*SID2*) were both upregulated in *old12* as compared to wild type (Fig. 6C). In addition, *PHYTOALEXIN DEFICIENT 4* (*PAD4*) and the SA modifier *BENZOIC ACID/SALICYLIC ACID METHYLTRANSFERASE 1* (*BSMT1*) also showed increased expression in *old12* as compared to wild type. NPR1 has been shown to act as a SA receptor that functions as a positive regulator of the SA signal transduction pathway [54]. Because *old12* shows spontaneous cell death it might act through NPR1. However, we found that the *old12 npr1-2* double mutant results in leaf bleaching (Fig. S8), similar to what had been previously reported for a cross between *old1*/*cpr5* and *npr1* [55]. As *npr1* mutants grown on SA show bleaching [54], it can be assumed that the observed phenotype is a consequence of high SA levels in *old12*. Subsequent crosses between *old12* plants and plants harbouring the *nahG* gene, which encodes a SA hydroxylase, were analyzed for the effect of reducing SA levels on the *old12* phenotype. By using genetic markers, an F2 population of *old12* x *nahG* plants was analyzed (Fig. 6D). Plants that were homozygous for the *old12* mutation showed stunted growth and early cell death, whereas those that also contain the *nahG* gene appeared normal. These results indicate that suppression of SA biosynthesis in the *old12* mutant is sufficient to repress the phenotype.

### The *old12* mutant shows enhanced resistance to *Pseudomonas* DC3000

**Fig. 7:**
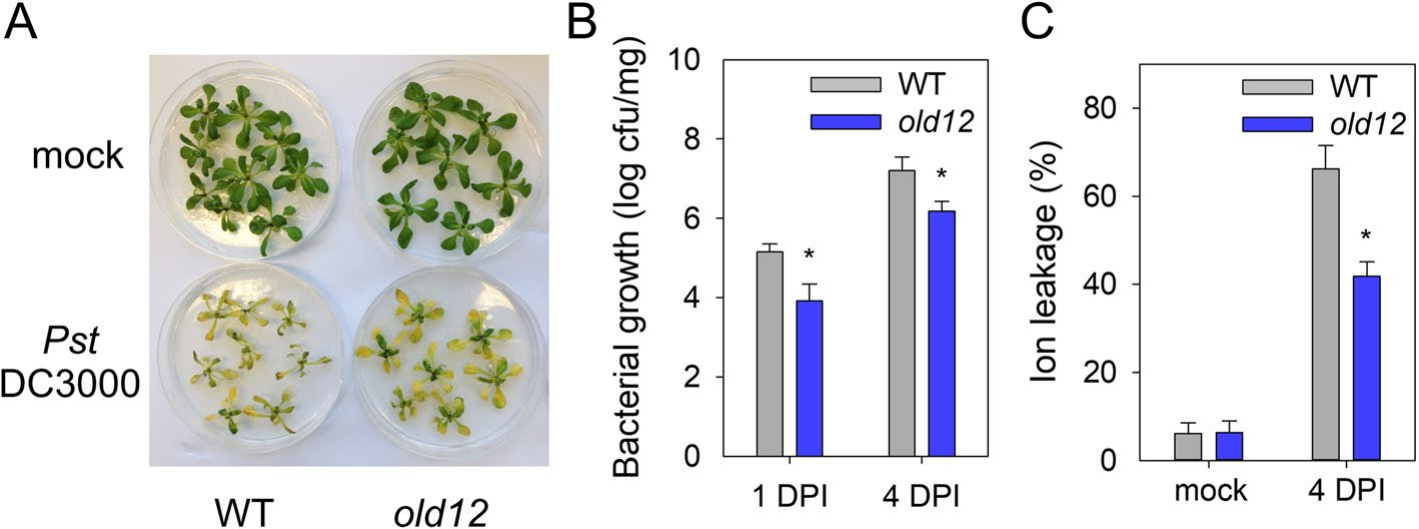
*old12* plants show increased resistance against *Pst* DC3000. **A** Seedlings were inoculated with water (Mock) or 5 × 10.^5^ bacteria/ml of *Pst* DC3000 for 4 days. Experiments were repeated twice and showed similar results. **B** The colony-forming units (cfu) of *Pst* DC3000 in *old12* are significantly different from WT after one day and four days post infection. Each column represents the mean ± SE from 6 biological replicates; the experiment was repeated twice and revealed similar results. Asterisks indicate significant difference between *old12* and wild-type plants (Student’s *t*-test; *p* ≤ 0.05). DPI: days-post-inoculation **C** Ion leakage of L*er*-0 and *old12* seedlings after 4 days of incubation with *Pst* DC3000. Data represent means ± SE from 6 biological replicates. (* *p* ≤ 0.05; Student's *t*-test)

Several LecRK members have been reported to be involved in plant defense. The mutant *lecrk-v.5* in L*er*-0 shows resistance to the plant pathogen *Pst DC3000* due to elevated ROS levels in guard cells [56, 57]. Although on soil an early leaf death phenotype is observed, when grown in vitro, the *old12* mutant is indistinguishable from wild type (Fig. 7). To test the effect of *old12* on plant susceptibility to *Pst* DC3000 we performed a flood inoculation assay [58]. Four days after inoculation, most rosette leaves of the wild type (WT) had wilted whereas *old12* plants appeared more vital (Fig. 7A). Subsequently, bacterial growth was analyzed and both after one and four days of inoculation with *Pst* DC3000 the bacterial growth in *old12* was reduced as compared to wild type (Fig. 7B). Furthermore, a higher percentage of ion leakage (an indicator of cell death) was observed in the wild type as compared to *old12* four days post inoculation (Fig. 7C). These results demonstrate that the *old12* mutation in *P2K2* enhances plant disease resistance to *Pst* DC3000, which is in line with the constitutive activation of defense responses.

## Discussion

Temperature has a profound impact on immune pathways in plants and their influence on growth and development [59]. While short exposure to warm temperatures enhances the output of the pattern-triggered immunity (PTI) response, effector-triggered immunity (ETI) responses that result in cell death are compromised under high temperatures [60, 61]. In line, many autoimmune phenotypes can be reversed by slight elevation in temperature [37]. Here, we characterized the early leaf death mutant *old12*, whose growth defect is recovered at high temperatures. The *old* mutants were originally isolated as altered leaf senescence mutants resulting in the concept of the senescence window [26]. The concept describes the leaf age dependent onset of senescence during developmental and precocious senescence. For *old12* it has been shown that the premature onset of senescence can not be enhanced by exogenously applied ethylene, suggesting that it might act independently from it, or downstream [6]. Here, we show that the *old12* mutant displays constitutive defense gene activation, localized cell death, enhanced SA biosynthesis gene expression, and improved resistance to *Pst* DC3000. In particular, we found that the *old12* mutant is caused by a point mutation in *P2K2*, belonging to a family of receptor-like kinases that so far have been associated with plant–microbe interactions and with responses to abiotic stress [62]. P2K2 has been previously identified as an extracellular ATP-sensing kinase, that can complement the function of P2K1 [33]. Still, P2K1 and P2K2 do seem to differ in their extracellular sensing domains as P2K1 was shown to detect the secreted *Phytophthora infestans* effector protein induced-O (IPI-O) through specific amino-acid residues that are not present in P2K2 [63]. Interestingly, we found that the induction of the autoimmune phenotype by the *old12* mutation occurs accession-specific.

Our molecular and genetic analyses indicate that elevated SA levels are responsible for the immune activation, cell death and growth defects observed in *old12*. In *old12*, SA biosynthesis and signaling genes are expressed at a higher level than in wild type. Arabidopsis plants grown under optimal conditions have low basal SA levels, that are rapidly elevated during pathogen infection through transcriptional upregulation of the biosynthesis pathway [64]. SA is known to be required for spontaneous cell death in several lesion-mimic mutants including *lsd6*, *lsd7*, *accelerated cell death 6* (*acd6*), and *acd11* mutants [28, 65]. Similar to the *acd11* mutant [28], the introduction of the *nahG* transgene into *old12* fully restores the growth defect and prevents precocious onset of leaf death. In addition, increased SA levels are also supported by crossing the *npr1* mutant with *old12*, resulting in a typical leaf bleaching that normally is observed when *npr1* mutants are treated with SA [54]. Several SA-related autoimmune mutants, including *sensitive to low humidity 1* [66], *old3* [38] and *copine-1* [67], can also be restored by growing them under high humidity. Here, we found that *in-vitro* cultivation of *old12* suppresses the early cell death phenotype. Still, not all lesion-mimic mutants require SA accumulation for activation of spontaneous cell death nor do high SA levels always cause cell death [68, 69]. Taken together, our analysis reveals that the observed *old12* autoimmune and growth retardation phenotype is SA-dependent. As the exact mechanism of how SA regulates cell death is still unclear in plants [19], elucidating the molecular trigger mechanism in *old12* might help to uncover novel regulatory proteins.

The Arabidopsis genome encodes 45 L-type Lectin receptor-like kinases (LecRKs), which have an extracellular L-lectin domain that is involved in sensing, and a cytosolic kinase domain for signal transduction [46]. To date, several L-type LecRKs have been demonstrated to be activated by pathogen-associated molecular patterns (PAMPs) to trigger PTI and other plant responses against pathogens [62]. LecRK-IX.2 has been found to be required for the activation of PTI responses upon flg22 treatment, including the activation of SA responses [70]. In addition, constitutive overexpression of *LecRK-IX.2* causes premature cell death and dwarfism in Arabidopsis [71], two phenotypic features we also observed for the *old12* mutation affecting P2K2. The most prominent member of the LecRKs is P2K1 which was shown to be a sensor for extracellular ATP [33, 34]. P2K1 is required for resistance against several pathogens, including *Phytophthora infestans* and *Pst* [72, 73]. Moreover, P2K1 detects an effector of *Phytophthora infestans* through its extracellular domain [63]. The resistance against *Pst* DC3000 by P2K1 relies on downregulation of the MYC2-mediated jasmonic acid (JA) pathway [74]. Recently, P2K2 was reported to be able to interact with P2K1 and contribute to plant defense against bacterial phytopathogen *P. syringae* [33]. Loss of function mutants of *P2K2* in the Col-0 accesion result in increased susceptibility. Here, we demonstrate that the *old12* mutation in P2K2 results in constitutive defense responses, which consequently enhances tolerance against *Pst* DC3000. So far, we did not study if overexpression of P2K2 might improve pathogen tolerance. Previous reports indicate that plants ectopically expressing P2K2 show an increased calcium flux upon eATP [33], suggesting that these lines might also respond stronger to pathogens.

*P2K2* expression is most notably in root tips but also weakly detectable in the aerial parts of the plant. In contrast, *P2K1* is strongly expressed in most tissues [46]. Although P2K2 has a higher affinity towards ATP than P2K1 [33], the tissue-specific expression of *P2K2* potentially renders P2K2 the primary eATP receptor in root tips. As *P2K1* is also expressed in the root apex [75], it is possible that in this tissue both receptors build a heteromeric complex as previously suggested [33]. Within this context it is interesting to note that roots, upon touching, release ATP to the extracellular space [76]. Potentially, P2K2 and P2K1 are involved in avoiding impenetrable obstacles (e.g. rocks in the soil) during root growth by participating in mechanosensing.

The *old12* mutation affects amino acid C407 in the kinase domain of P2K2. In this study, we revealed that a mutation in this residue results in an impaired in vitro kinase activity. Potentially, the reduced kinase function by the *old12* mutation results in the autoimmune responses observed. That said, loss-of-function alleles in Col-0 do not affect the normal growth phenotype, and no constitutive activation of defense genes is observed [33]. The C407 residue is conserved among receptor-like kinases, and for P2K1 it was shown that it is S-acylated upon ATP sensing [77]. As a result, a negative feedback regulation is established that dampens the immune response. Moreover, ATP-induced resistance to the virulent bacterium *Pst* DC3000 is improved in *p2k1* mutants complemented with the P2K1^C407A^ mutation as compared to wild type [77]. Although it is not clear if also the kinase activity of P2K1 was affected, it might explain why the *old12* mutation in P2K2 also results in improved resistance against *Pst* DC3000. Still, complemented P2K1^C407A^ lines do not cause autoimmunity, suggesting that additional mechanisms in the L*er*-0 background are activated.

The autoimmune response caused by the *old12* mutation occurs accession-specifically. We noticed that in L*er*-0, Nd-0 and C24 the phenotype solely depends on the C407Y mutation in P2K2, however, in Col-0, Ct-1 and Tsu-0, an additional recessive allele is required for the phenotype to occur. Moreover, the L*er*-0 allele has a 22-amino acid C-terminal extension, which is conserved amongst P2K2 allelles of *Brassicaceae* and might be essential for the autoimmune phenotype observed. We suggest that both the C-terminal extension as well as an accession specific locus on chromosome I are required for the phenotype to occur in Col.

## Conclusions

We identified the first autoimmune mutant in Arabidopsis caused by a mutation in a LecRK family member. The *old12* mutation affects a conserved cysteine residue that recently has been shown to be S-acylated in P2K1 to dampen the immune responses triggered by eATP receptors. The phenotype observed is fully reversible in a temperature- and/or SA-dependent manner. Further work unveiling the triggering mechanisms of the autoimmune phenotype will help to understand the function of the C-terminally extended P2K2 allele. As the allele is conserved amongst agronomically important crops, unravelling the mechanism by which P2K2 triggers PCD represents a valuable future research avenue to follow.

## Methods

### Plant material and growth conditions

The *old12* mutant was obtained from an EMS–mutagenized *Arabidopsis thaliana* L*er*-0 collection [6]. In addition to L*er*-0, also Col-0, Ct-1, Nd-0, C24 and Tsu-0 obtained from the Nottingham European Arabidopsis Stock Center (NASC, http://arabidopsis.info) were used. *NahG* (in L*er*-0) and *npr1-1* were obtained from the Xin-Nian Dong lab [54]. Arabidopsis plants were grown on soil (Einheitserde Typ VM; Einheitserdewerke Werkverband e.V., Sinntal-Altengronau, Germany) with a 21 °C day/16 °C night regime, and a photoperiod of 16 h light (120 μmol⋅m^−2^⋅s^−1^) and 8 h dark (long-day condition), or a photoperiod of 8 h light and 16 h dark (short-day condition). For in vitro growth, seeds were sown on half-strength Murashige and Skoog (MS) medium containing 0.5% (wt/vol) sucrose, stratified for 48 h at 4 °C, and germinated at 21 °C day/16 °C night with a photoperiod of 16 h light (140 μmol⋅m^−2^⋅s^−1^) and 8 h dark.

### Senescence scoring, chlorophyll content and ion leakage

Cotyledons or rosette leaves with over 10% yellow leaf area were judged as senesced [6]. Chlorophyll content was measured either according to Inskeep and Bloom [78] by extraction in DMF overnight at 4 °C in darkness and subsequent spectrophotometric quantification, or by using a SPAD analyser (N-tester, Hydro Agri Imminham, UK) according to Levey and Wingler [79]. Leaves used for chlorophyll extraction were imaged to determine the leaf area.

Ion leakage was measured as previously described [26]. In short, leaf samples were immersed into deionised carbonate-free water, shaken in a 25 °C water bath for 30 min, and the conductivity was measured using an ion conductivity meter (B-173; Horiba, Tokyo, Japan). Subsequently, samples were boiled for 10 min and conductivity was measured again. Ion leakage was calculated as the percentage of the first measurement over the second measurement.

### Callose, cell death and GUS staining

For callose staining, the second pair of rosette leaves of 3-week-old plants was collected and.destained in 95% EtOH, and stained with aniline blue as described previously [80]. Staining in 0.05% aniline blue was performed overnight in 0.07 M phosphate buffer (pH 9.0). Leaves stained with aniline blue were imaged with an epifluorescence microscope with UV filter. Cell death was visualized using trypan blue staining [81].

Whole leaves were boiled for 1 min in the staining solution and then decolorized in chloral hydrate for at least 30 min. Leaves were photographed by using a stereo-microscope (Leica).β-Glucuronidase activity was detected histochemically by 5-bromo-4-chloro-3-indolyl-β-D-glucuronic acid (X-Gluc) [82]. Tissues were submerged in GUS staining solution and incubated overnight at 37℃. Tissues were cleared in 70% ethanol and subsequently examined and imaged using a stereo-microscope (Leica).

### Constructs and plant transformation

For in vitro expression of the P2K2 kinase domain the pF3A WG (BYDV) vector (Promega) was used with a 1185-bp C-terminal cDNA fragment of the *P2K2* coding region, that was amplified with the following primers: 5'-TTTGCGATCGCATGGATTACAAGGATGACGATGACAAGGCAGCCGGTGCTGTTCTTGCAGGAGTTTATT-3' and 5'-AAAGTTTAAACTTACCTGTCACTGTTTAGCTTAGAGA-3' from either L*er*-0 or *old12* cDNA. For constructing a promoter-GUS reporter for *P2K2*, a 2117-bp gDNA fragment from L*er*-0 was amplified using the primers 5'-CACCCAATTATCCATGGGGATGGACG-3' and 5'-TGCTGCTGATGAAACAGAGAG-3'. By using the same primers on Col-0 gDNA a 1951-bp upstream fragment was amplified that was also used for creating a promoter-GUS reporter for the Col-0 allele. After ligation into pENTR/D-TOPO vector (Invitrogen) the promoter was recombined into the pKGWFS7.0 binary vector [83] using LR clonase (Invitrogen). To complement the *old12* mutant, a genomic DNA fragment of 4575-bp containing the wild-type *P2K2* coding region, the upstream promoter and downstream terminator, was cloned from L*er*-0. The DNA fragment was amplified by PCR using the primers 5'-CACCATTGTGCTCCCCTTGTGAAG-3' and 5'-TTATGGAAGCGCACGTGTAG-3', and cloned into the pENTR/D-TOPO vector. Subsequently the entry clone was recombined with the pKGW binary vector [83]. For localization studies in protoplast, the full-length CDS of *P2K2* was amplified from cDNA with the following primers: 5'-CACCATGTCTGAAGGATTGTTTCTGTTCTG-3' and 5'-CCTGTCACTGTTTAGCTTAGAGA-3'. After cloning into pENTR/D-TOPO the CDS was recombined into p2GWY7 [83], resulting in a C-terminal fusion with the YELLOW FLUORESCENT PROTEIN (YFP).

The binary vectors for the GUS reporter and complementation test were transformed into *Agrobacterium tumefaciens* strain GV3101 by electroporation [84]. Subsequently, plants were transformed using floral dip [85].

### Next-generation mapping

The *old12* mutant was crossed to WT Col-0 to generate a mapping population. F_2_ plants showing the *old12* mutant phenotype (157 plants) were pooled and used for genomic DNA isolation by using the CTAB method [86] followed by whole-genome sequencing. Whole-genome sequencing was carried out by using 100 nt paired-end sequencing (> 50 × coverage for all DNA samples) on an Illumina GA IIx platform. The sequence data was aligned against the Arabidopsis Col-0 TAIR10 reference sequence using bowtie2 [87], variant calling was done using samtools [88] and assessed by a custom script following the methods of next-generation mapping [41] and SHOREmap [40].

### RNA isolation and qPCR analysis

Total RNA was extracted from leaves or seedlings using the RNeasy kit (Qiagen) according the manufactures recommendations. RNA samples were DNase I treated (Invitrogen) prior to cDNA synthesis with the Thermo Scientific RevertAid First Strand cDNA Synthesis Kit. Quantitative real-time PCR was done using PowerUp SYBR Green PCR Master Mix (Applied Biosystems). *ACTIN2* (AT3G18780) was used as reference gene. Oligonucleotides for qPCR were designed with the webtool QUANTPRIME [89] (Table S2). Relative transcript abundance was determined by the comparative C_T_ method [90].

### Flood-inoculation assay

Flood-inoculation assays were performed essentially as previously described [58], with some small modifications. Two-week-old plants grown on half-strength MS plates were inoculated with 5 × 10^6^ cfu/ml *Pseudomonas syringae* pv. tomato DC3000 (*Pst* DC3000) containing 0.025% Silwet L-77 for 2–3 min at room temperature. The bacterial suspension was removed by decanting, and plates were sealed and incubated under long-day conditions. In each experiment, six samples were evaluated, and each experiment was repeated at least two times. To determine the bacterial growth in Arabidopsis leaves, whole rosettes were collected from the plate and the total weight was measured. Subsequently, the rosette leaves were surface-sterilized with 75% EtOH for 2 min. After washing three times with sterile distilled water, the samples were homogenized in 500 µl sterile distilled water using a mortar and pestle, and diluted samples were plated onto KBM medium containing 100 mg/l rifampicin antibiotic. Two days after incubation the number of colony-forming units (CFU) was determined.

### In vitro protein expression, protein kinase assays and immunoblotting

In vitro expression of the P2K2 kinase domain was performed by using 4.0 µg of the pF3A WG (BYDV) plasmid encoding a FLAG-tagged kinase domain in the TNT SP6 High Yield Wheat Germ Mastermix (Promega). The FLAG-tagged protein was cleaned-up by binding to Anti-FLAG® M2 Magnetic Beads (Sigma) and washing three times in 50 mM Tris–HCl pH 7, 50 mM NaCl. After the final wash, protein was eluted in 50 µl 1X kinase buffer A (40 mM Tris–HCl (pH 7.5), 20 mM MgCl) containing 3X FLAG® Peptide (Sigma). Amount of purified protein was estimated by performing immunoblotting using an anti-FLAG tag antibody (Sigma). Substrate-labeling reactions were essentially performed as described [91]. In brief, 20-µl kinase reactions were performed by using up to 10 µl purified P2K2 kinase domain in Kinase Buffer A containing 1 mM ATPγS (Sigma Aldrich), and in the case of transphosphorylation assay 1 µg MBP (Invitrogen) was included. After 60 min, the kinase reaction was stopped by adding 20 mM EDTA and the thiophosphorylated substrate was alkylated with 2.5 mM PNBM (Abcam) in 5% (v/v) DMSO for 2 h. The alkylation reaction was mixed with SDS loading buffer before samples were subjected to SDS-PAGE and transferred to a nitrocellulose membrane (Amersham). Immunodetection was performed using rabbit monoclonal antibodies against the thiophosphate ester (α-TPE; Abcam) to detect thiophosphorylated kinase substrates. Rabbit α-FLAG (Sigma) served to check for equal gel loading. Antigen–antibody complexes were detected with infrared (IR)-labeled anti-rabbit secondary antibodies (LiCOR). Using independent protein expression reactions, all substrate thiophosphorylation reactions were repeated at least twice with comparable results.

### Accessions numbers

Gene IDs involved in this paper are as follows: *P2K1* (AT5G60300), *P2K2* (AT3G45430), *LecRK-I.10* (AT5G60310), *BAK1* (AT4G33430), *CERK1* (AT3G21630), *ICS1* (AT1G74710), *PAD4* (AT3G52430), *EDS1* (AT3G48090), *PR1* (AT2G14610), *PR2* (AT3G57260), *PR5* (AT1G75040), *SAG13* (AT2G29350), *SAG101* (AT5G14930), *SAG113* (AT5G59220), *NPR1* (AT1G64280), *NPR2* (AT4G26120), *MES1* (AT2G23620), *BSMT1* (AT3G11480) and *SID2* (AT1G74710).

## Supplementary Information

**Additional file 1:** **Fig. S1.**  Early-senescence phenotype of *old12* under short-day condition. **Fig. S2.** Visualization of allele frequency by SHOREmap. **Fig. S3.** Absence of *old12* phenotype in *p2k2-1*. **Fig. S4.** Comparison of the protein sequence of P2K2 from different accessions. **Fig. S5.** Conservation of the C-terminal extension of P2K2 in *Brassicaceae*. **Fig. S6.** Full blot images for figure 4 **Fig. S7.** Tissue-specific expression of *P2K2* in Col-0. **Fig. S8.** Crossing of *npr1* into the *old12* mutant results in leaf bleaching. **Table S1.** Putative EMS-induced SNPs on Chromosome I. **Table S2.** qPCR primers used.

## Data Availability

All relevant data can be found within the manuscript and its supporting material. Lines used within the study can be requested for at the corresponding author.

## Abbreviations

OLD: Onset of Leaf Death
SAG: Senescence associated gene
PCD: Programmed cell death
HR: Hypersensitive response
SA: Salicylic acid
PR: Pathogenesis-related
NB-LRR: Nucleotide‐binding leucine‐rich repeat
LecRK: Lectin receptor kinase
RLK: Receptor-like kinase
DAS: Days after sowing
MBP: Myelin basic protein
