# Supplementary material for: The Arabidopsis thaliana onset of leaf death 12 mutation in the lectin receptor kinase P2K2 results in an autoimmune phenotype
Source: BMC Plant Biol. 2023 Jun 2;23:294. doi: 10.1186/s12870-023-04300-0 (PMC10236711; doi:10.1186/s12870-023-04300-0)
Supplement: Supplementary file 1 — Additional file 1: Fig. S1. Early-senescence phenotype of old12 under short-day condition. Fig. S2. Visualization of allele frequency by SHOREmap. Fig. S3. Absence of old12 phenotype in p2k2-1. Fig. S4. Comparison of the protein sequence of P2K2 from different accessions. Fig. S5. Conservation of the C-terminal extension of P2K2 in Brassicaceae. Fig. S6. Full blot images for figure 4 Fig. S7. Tissue-specific expression of P2K2 in Col-0. Fig. S8. Crossing of npr1 into the old12 mutant results in leaf bleaching. Table S1. Putative EMS-induced SNPs on Chromosome I. Table S2. qPCR primers used. [file 12870_2023_4300_MOESM1_ESM.docx]

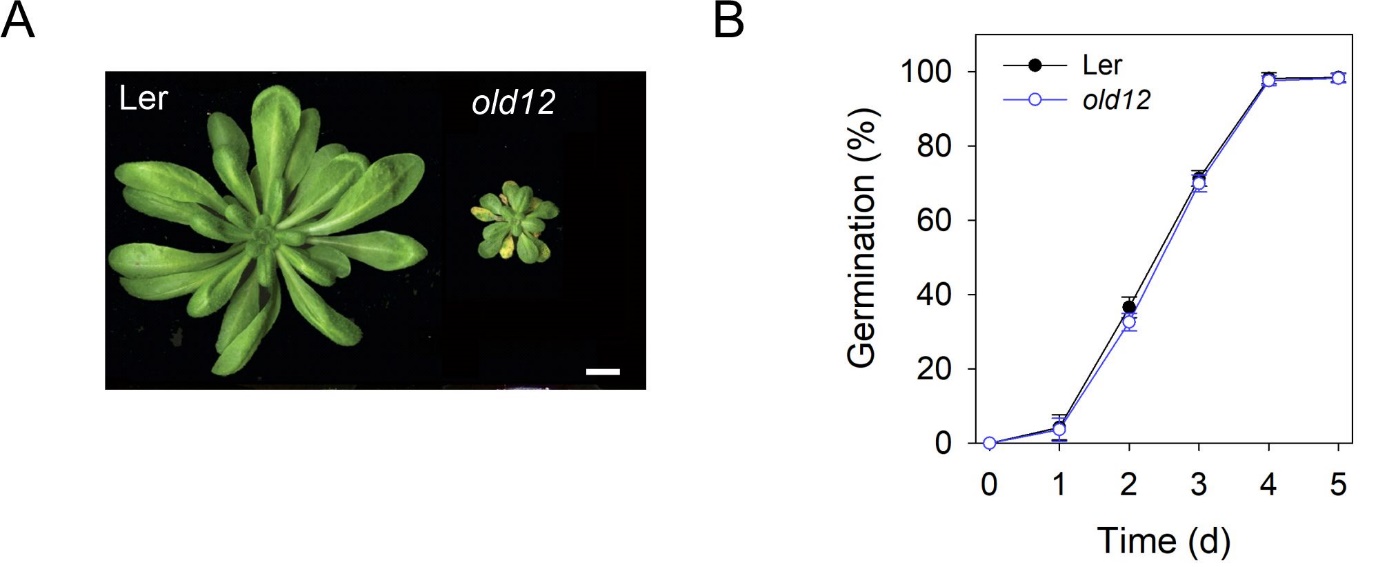


**Fig. S1** Early-senescence phenotype of *old12* and germination efficiency. **A** Shown are a wild-type plant (L*er*-0; left) and the *old12* mutant (right), grown under short-day condition, at 48 DAS. **B** Seeds of the *old12* mutant and wild type (Ler) were assayed for their ability to germinate on water. Data are means ± SD (n = 4).


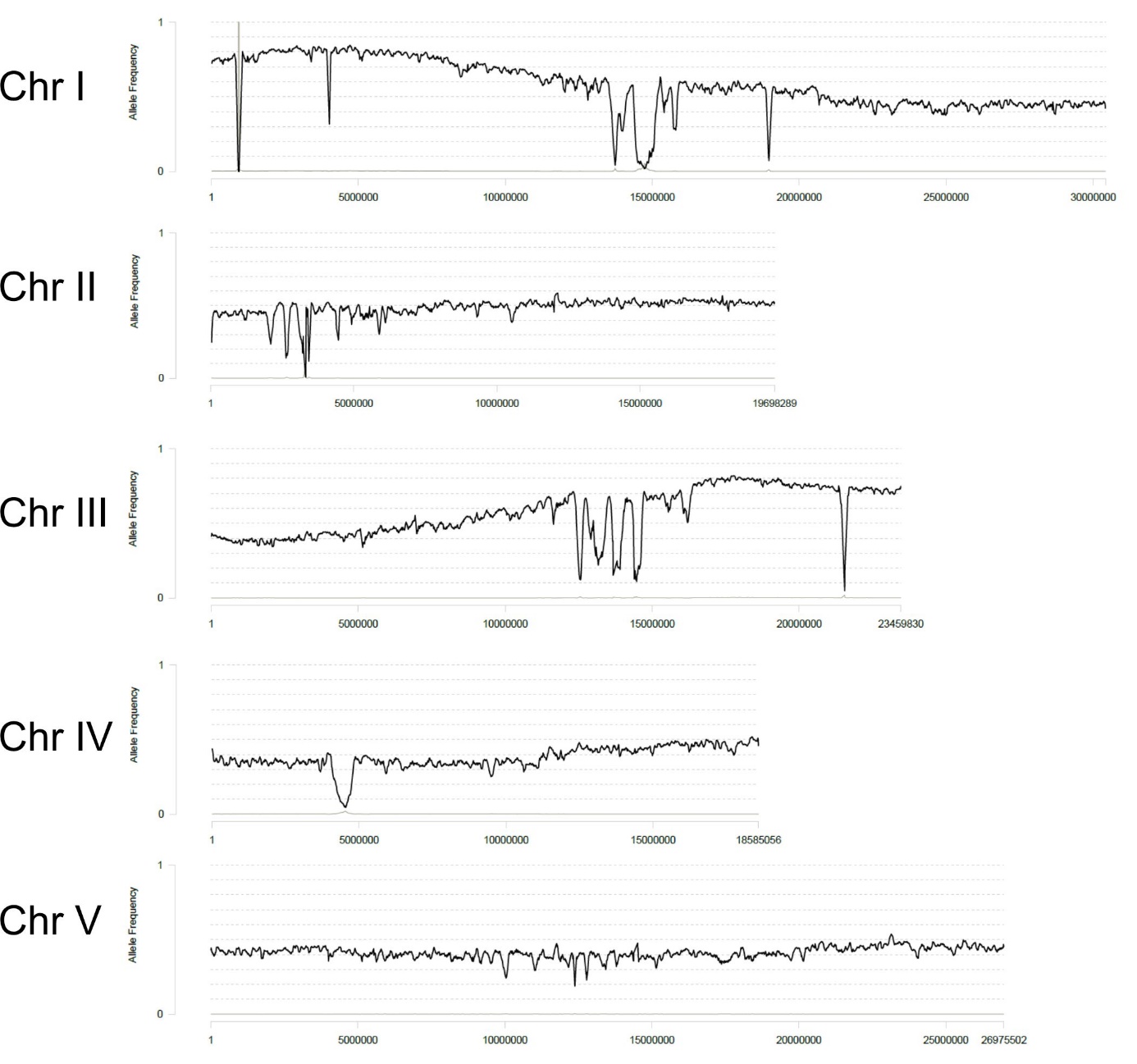


**Fig. S2** Visualization of allele frequency by SHOREmap**.** Visual output from the SHOREmap analysis on the sequencing data obtained with the *old12* mapping population. The black lines indicate relative parental allele frequencies in a sliding window of 100 kb. Both, on chromosome I and on chromosome III an enrichment of the L*er*-0 parental DNA is observed, indicating that the *old12* phenotype relies on two alleles in the mapping population.

**
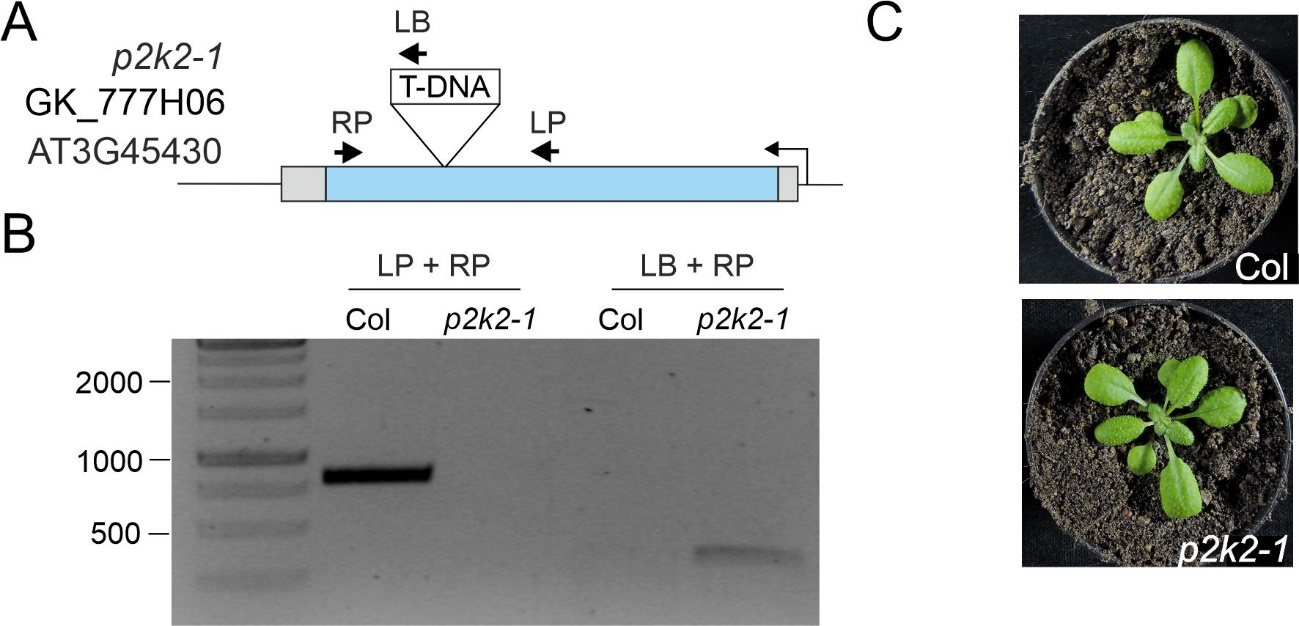
**

**Fig. S3** Absence of the *old12* phenotype in *p2k2-1***.** (a) Identification of the T-DNA insertion line GK_777H06, *p2k2-1*. The diagram illustrates the relative position of the T-DNA insertion in the coding sequence of AT3G45430. The arrow indicates the direction of transcriptional start site. Primers used for genotyping are indicated (LP, RP and LB) (b) Genotyping of T-DNA insertion line. Genomic DNA isolated from either the wild type (Col) or the *p2k2-1* (GK_777H06) mutant was isolated and used for a PCR reaction. The LP + RP primer pair results in a specific product from the *P2K2* locus. The LB + RP primer pair resulted only in a product from the T-DNA insertion line (c) Representative pictures of 27 DAS wild-type and *p2k2-1* mutant plants grown under long-day condition.


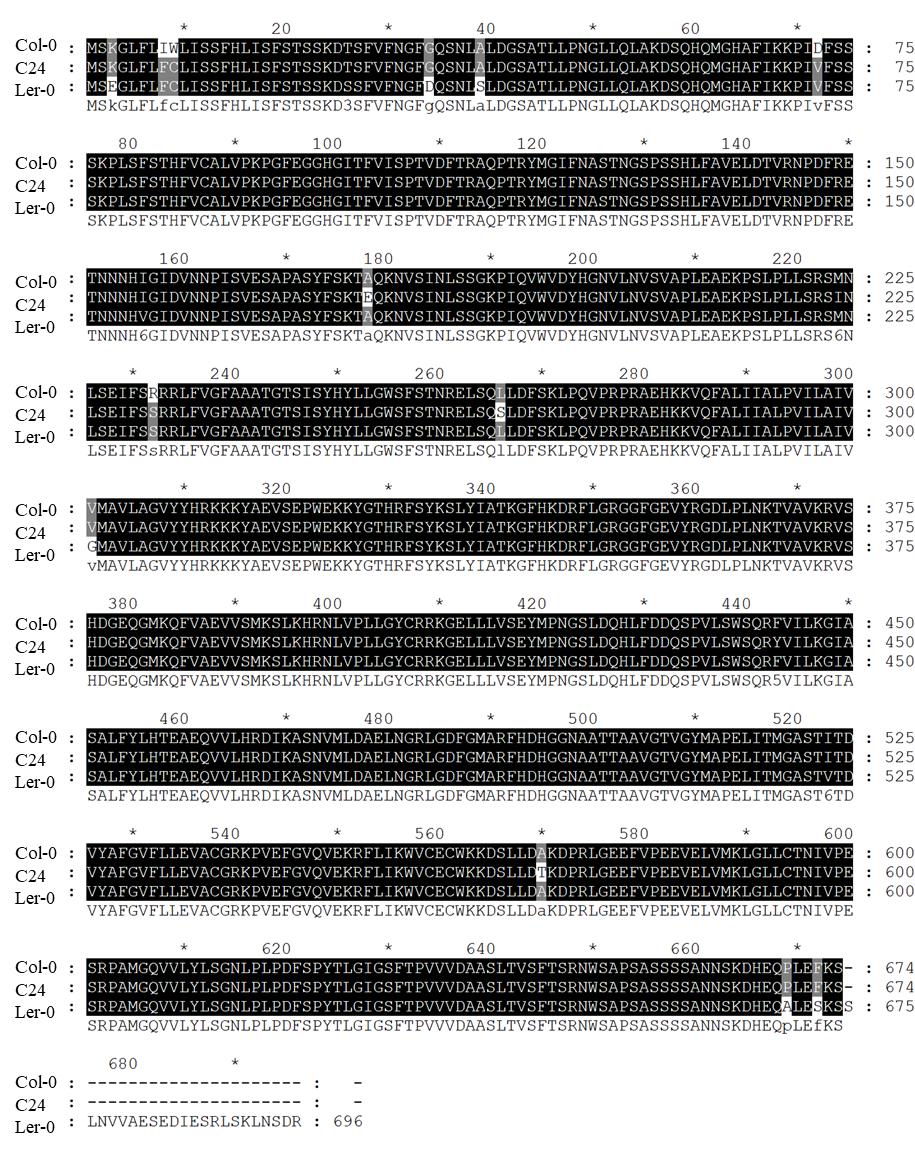


**Fig. S4** Comparison of the protein sequence of P2K2 from different accessions. Protein sequences of P2K2 from L*er*-0, Col-0 and C24 were aligned using ClustalX (http://www.clustal.org/) and visualized with GeneDoc (https://genedoc.software.informer.com/ 2.7/). Black-shaded residues are conserved. The L*er*-0 protein has a 22-amino acid extension at its C-terminus.


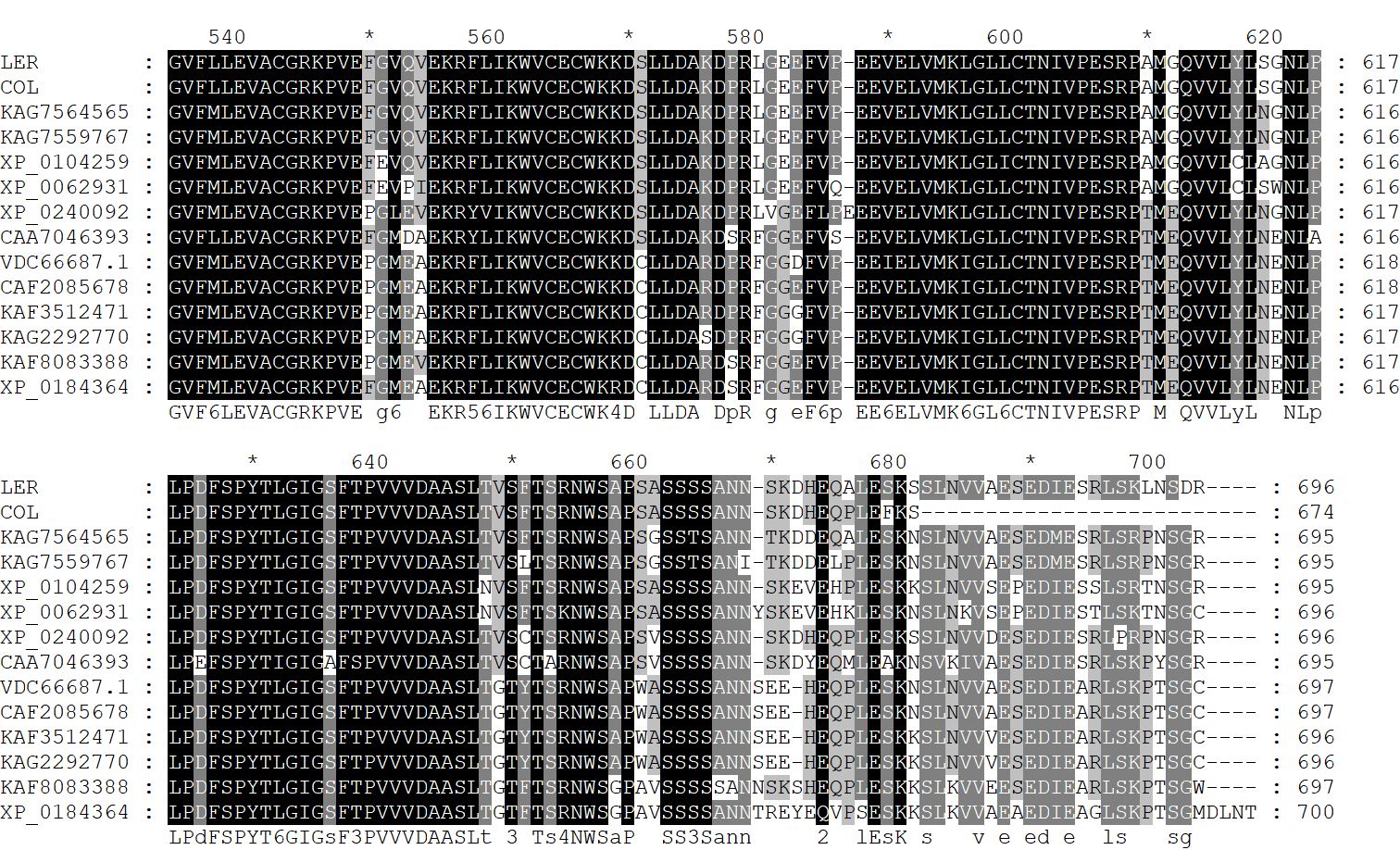


**Fig. S5** Conservation of the C-terminal extension of P2K2 in *Brassicaceae*. Protein sequences of P2K2 from different Brassicaceae were aligned using ClustalX (http://www.clustal.org/) and visualized with GeneDoc (https://genedoc.software.informer.com/2.7/). Black-shaded residues are conserved. The protein IDs represent genebank IDs. KAG7564565.1 *Arabidopsis suecica*; KAG7559767.1 *Arabidopsis arenosa*; XP_024009224.1 *Eutrema salsugineum*; VDC66687.1 *Brassica rapa*; CAF2085678.1 *Brassica napus*; KAF3512471.1 *Brassica cretica*; KAG2292770.1 *Brassica carinata*; CAA7046393.1 *Microthlaspi erraticum*; XP_010425969.1 *Camelina sativa*; KAF8083388.1 *Sinapis alba*; XP_018436484.1 *Raphanus sativus*; XP_006293115.1 *Capsella rubella*.

**
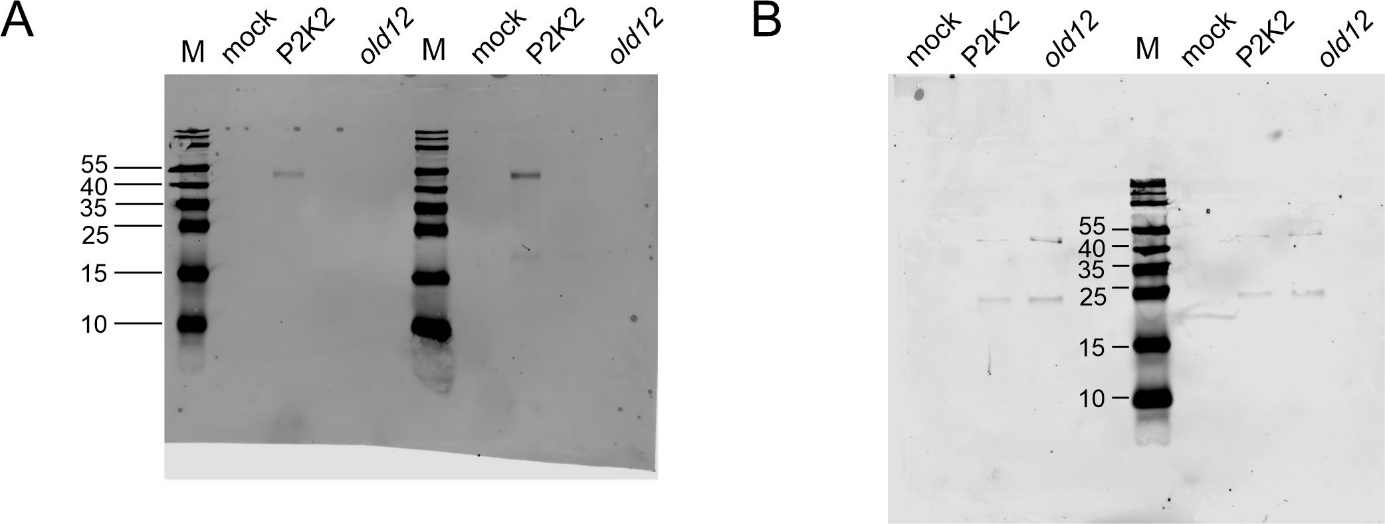
**

**Fig. S6** Full blot images for figure 4. (a) Uncropped P2K2 autophosphorylation and transphosphorylation blot for the results shown in main figure 4. Ladder and bands detected with an antibody against the thiophosphate ester are shown. (b) Uncropped loading control blot for the used purified kinase domains of wild-type P2K2 and *old12*. Protein bands were detected using an anti-FLAG antibody.

**
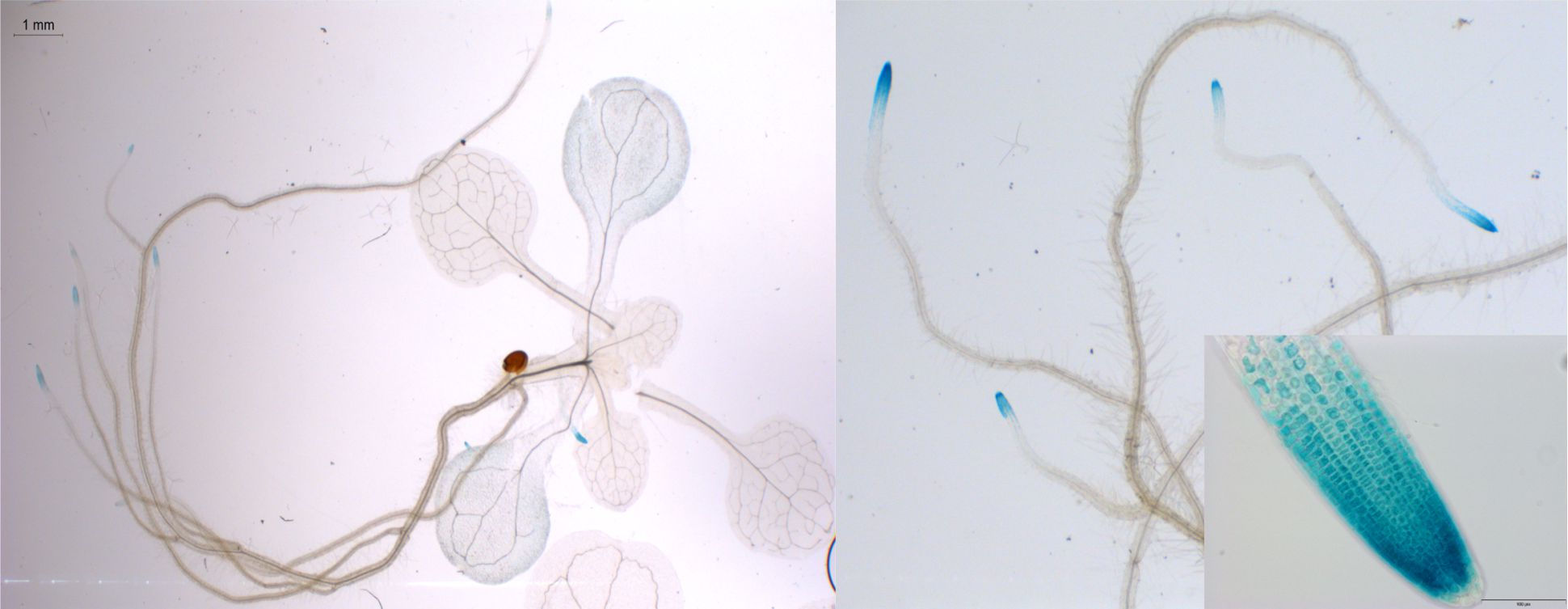
**

**Fig. S7** Tissue-specific expression of *P2K2* in Col-0. A 1951-bp upstream promoter fragment of *P2K2* was cloned from Col-0 to drive a *GUS* reporter. Visualization of GUS activity revealed that *P2K2* is mainly expressed in root tips, similar results were obtained with the promoter derived from L*er*-0 (Figure 5).


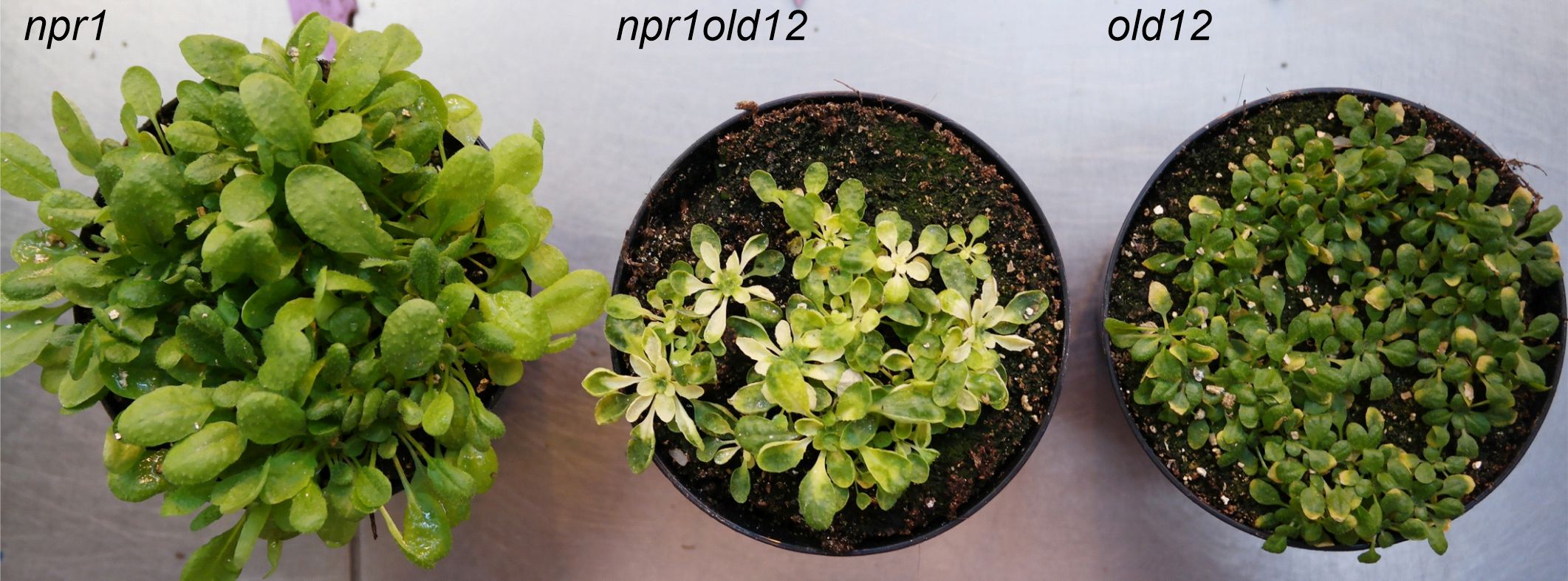


**Fig. S8** Crossing of *npr1* into the *old12* mutant results in leaf bleaching. Shown are *npr1*, *npr1old12* and *old12* plants grown under long-day condition for 4 weeks. Introduction of the *npr1* allele into *old12* results in bleaching of the leaves, while plant size remains largely unaffected.

**Table S1: Putative EMS-induced SNPs on Chromosome I**

| **Position** | **Col** | ***old12*** | **Gene** | **Effect** |
| --- | --- | --- | --- | --- |
| 2311486 | C | T | AT1G07520 | S107N |
| 3127037 | G | A | AT1G09650 | R91K |
| 3729242 | G | A | AT1G11150 | R91K |
| 4141812 | G | A | AT1G12210 | V289I |
| 4142349 | G | A | AT1G12210 | A468T |
| 4548535 | G | A | AT1G13280 | A250T |
| 5137582 | G | A | AT1G14890 | V180I |
| 5371549 | C | T | AT1G15610 | P192L |

**Table S2:** **qPCR primers used**.

| Name | AGI code | 5’-3’ Forward | 5’-3’ Reverse |
| --- | --- | --- | --- |
| NPR2 | AT4G26120.1 | ACTCCAGAGGAGTTGAGGATGAGG | AGAAGTCGAGCAAGTGCAACTC |
| NPR1 | AT1G64280 | AACGATTCTTCCCGCGCTGTTC | TTCTCCGCAAGCCAGTTGAGTC |
| SAG113 | AT5G59220 | CGGGTGGTCGTGTTATCTACTG | CCTCCGGTCTGCTGATTACATAC |
| PR-1 | At2g14610 | AGGCACGAGGAGCGGTAGG | CATGTTCACGGCGGAGACG |
| PR-2 | At3g57260 | TACGGGATGCTAGGCGATACC | CTGGAGGCGAGACGTTCAAGAT |
| PR-5 | At1g75040 | TCGGCGATGGAGGATTTGAA | AGCCAGAGTGACGGGAGGAAC |
| SAG13 | At2g29350 | CAGCTTGCCCACCCATTGTTA | TGTTGTCGCTCGCCCATTC |
| SAG101 | AT5G14930 | ATGGAGTCTTCTTCTTCACT | CTCTACTTCTTCAACTACTG |
| PAD4 | At3g52430 | GTTCTTTTCCCCGGCTTATC | CGGTTATCACCACCAGCTTT |
| ACTIN2 | AT3G18780 | CCCGCTATGTATGTCGC | AAGGTCAAGACGGAGGAT |
| SID2 | AT1G74710 | GCTTGGCTAGCACAGTTACAGC | CACTGCAGACACCTAATTGAGTCC |
| BSMT1 | AT3G11480 | ACTCCATTGGCTCTCTAAGGTTCC | AGTAGGCCTTGTATGCACTTTGAG |
| MES1 | AT2G23620 | TGGTCACAGCTTTGGTGGCTTG | AGGCATGTTGCTTCCAAACTTGTC |
